# Transcriptome analysis of *Vibrio parahaemolyticus* in type III secretion system 1 inducing conditions

**DOI:** 10.3389/fcimb.2014.00001

**Published:** 2014-01-20

**Authors:** Seth D. Nydam, Devendra H. Shah, Douglas R. Call

**Affiliations:** ^1^Department of Veterinary Microbiology and Pathology, Washington State UniversityPullman, WA, USA; ^2^Paul G. Allen School for Global Animal Health, Washington State UniversityPullman, WA, USA

**Keywords:** *Vibrio parahaemolyticus*, type III secretion system, T3SS, RNA-seq

## Abstract

*Vibrio parahaemolyticus* is an emerging bacterial pathogen capable of causing inflammatory gastroenteritis, wound infections, and septicemia. As a food-borne illness, infection is most frequently associated with the consumption of raw or undercooked seafood, particularly shellfish. It is the primary cause of *Vibrio*-associated food-borne illness in the United States and the leading cause of food-borne illness in Japan. The larger of its two chromosomes harbors a set of genes encoding type III section system 1 (T3SS1), a virulence factor present in all *V. parahaemolyticus* strains that is similar to the *Yersinia ysc* T3SS. T3SS1 translocates effector proteins into eukaryotic cells where they induce changes to cellular physiology and modulate host-pathogen interactions. T3SS1 is also responsible for cytotoxicity toward several different cultured cell lines as well as mortality in a mouse model. Herein we used RNA-seq to obtain global transcriptome patterns of *V. parahaemolyticus* under conditions that either induce [growth in Dulbecco's Modified Eagle Medium (DMEM) media, *in trans* expression of transcriptional regulator *exsA*] or repress T3SS1 expression (growth in LB-S media, *in trans exsD* expression) and during infection of HeLa cells over time. Comparative transcriptomic analysis demonstrated notable differences in the expression patterns under inducing conditions and was also used to generate an expression profile of *V. parahaemolyticus* during infection of HeLa cells. In addition, we identified several new genes that are associated with T3SS1 expression and may warrant further study.

## Introduction

*Vibrio parahaemolyticus* (*V. parahaemolyticus*) is an emerging food-borne bacterial pathogen that has caused significant disease in several countries. It is the leading cause of *Vibrio*-associated food-borne illness in the United States and the leading cause of food-borne illness in Japan (Yeung and Boor, 2004; Su and Liu, 2007). Infection is most commonly acquired by the consumption of contaminated raw or undercooked seafood, shellfish in particular, and frequently results in gastroenteritis (Yeung and Boor, 2004). This organism possesses numerous virulence factors that include two phylogenetically distinct type III secretion systems (T3SSs) located separately on each of its two chromosomes and referred to as T3SS1 and T3SS2, respectively. Many strains also encode two thermostable direct haemolysins (TDHs) (Makino et al., 2003). The T3SS is a organelle possessed by several species of Gram-negative bacteria that mediates the transport of specialized proteins, termed “effectors,” directly from the bacterial cytoplasm into the cytosol of eukaryotic cells (Galán and Wolf-Watz, 2006; Coburn et al., 2007). A T3SS is comprised of a basal body that spans the bacterial inner and outer membranes, a needle complex that extends distally from the bacterial surface and mediates the passage of proteins between the bacteria and eukayotic cells, and a translocon apparatus that forms a pore in the eukaryotic cell membrane (Izoré et al., 2011).

T3SS1 is related to the Ysc family of T3SS injectisomes and is presumptively present in all strains of *V. parahaemolyticus* (Makino et al., 2003; Troisfontaines and Cornelis, 2005; Okada et al., 2010). Although not directly involved in gastroenteritis, the T3SS1 clearly causes cytotoxicity *in vitro* in multiple cells lines (Ono et al., 2006; Burdette et al., 2008; Zhou et al., 2009; Hiyoshi et al., 2010) and mouse mortality *in vivo* when delivered into the intraperitoneal or the intrapulmonary spaces (Hiyoshi et al., 2010; Piñeyro et al.,^2010^). There are currently three known effector proteins that are translocated into cells by T3SS1. These include VopS (*vp1686*), which AMPylates Rho-family GTPases and produces a cell rounding phenotype during infection (Casselli et al., 2008; Yarbrough et al., 2009), VopQ (VepA, *vp1680*), which induces PI3-kinase independent autophagy by creating gated lysosomal channels (Burdette et al., 2009; Matsuda et al., 2012; Sreelatha et al., 2013), and VPA0450, an inositol phosphatase that disrupts the integrity of the host cell membrane (Broberg et al., 2010). Although the structural components of the T3SS are often conserved between bacterial genera (Cornelis, 2006; Galán and Wolf-Watz, 2006), effector proteins are substantially more variable. This was highlighted in *V. parahaemolyticus* by the discovery of the T3SS1 effector protein VPA0450. This effector is not only encoded outside of its associated cluster of T3SS1 genes but also on a separate chromosome.

Transcription of T3SS1 genes is controlled by the AraC-like transcriptional activator ExsA (*vp1699*) and the anti-activator ExsD (*vp1698*), which interacts directly with ExsA to suppress transcription (Zhou et al., 2008). ExsD interacts with an “anti-anti-activator” ExsC (*vp1701*), which is bound by another protein, ExsE (*vp1702*). ExsE is necessary for adhesion with host cells and when ExsE is secreted from the cell, this presumably allows ExsC to bind and inhibit ExsD, freeing ExsA to upregulate T3SS1-related genes (Kodama et al., 2010; Zhou et al., 2010; Erwin et al., 2012). Previous studies have shown that T3SS1 activity can be induced by *in trans* expression of *exsA*, as well as by contact with HeLa cells and growth in Dulbecco's Modified Eagle Medium (DMEM) (Zhou et al., 2008).

Microarray analysis has been used previously to examine the transcriptional profiles of the T3SS in *V. parahaemolyticus* and have helped elucidate the effects of calcium and iron on swarming and T3SS1 expression (Gode-Potratz et al., 2010) in addition to the effects of bile salts on T3SS2 expression (Gotoh et al., 2010). Nevertheless, understanding of the components involved in activation, regulation and pathogenicity of T3SS1 remains incomplete. The recent advent of high-throughput sequencing technologies have made it possible to obtain detailed transcriptomic profiles of several bacterial pathogens (Passalacqua et al., 2009; Perkins et al., 2009; Beaume et al., 2010; Filiatrault et al., 2010; Pinto et al., 2011). In this study we employed RNA-seq methods to obtain global transcriptome patterns of *V. parahaemolyticus* under conditions that induce (growth in DMEM, *exsA* expression) and repress (growth in LB-S, *exsD* expression) activity of T3SS1 as well as during HeLa cell infection over time in a minimal media (Hank's Balanced Salt Solution). Through differential expression analysis of these transcriptional profiles we aim to broaden our understanding of T3SS1-related gene expression and to identify additional genes that are associated with T3SS1 function.

## Materials and methods

### Bacterial strains, plasmids, and growth conditions

*V. parahaemolyticus* strain NY-4 (serotype O3:K6) was the model for this study and plasmid constructs derived from this strain were developed previously (Zhou et al., 2008). Bacteria were cultured at 37°C with shaking in Luria-Bertani (LB) medium (Difco) supplemented with 2.5% (w/v) NaCl (LB-S) and ampicillin (100 μg ml^−1^) for non-plasmid bearing strains (the wild-type NY-4 strain is ampicillin resistant), and with ampicillin (100 μg ml^−1^) and chloramphenicol (34 μg ml^−1^) for plasmid bearing strains unless otherwise specified. HeLa (ATCC^®^ CCL-2™) cells were maintained at 37°C with 5% CO_2_ in DMEM (Thermo Scientific, Catalog #SH30022.01) supplemented with 10% (v/v) fetal bovine serum (FBS) (Atlanta Biologicals, Catalog #S11150).

### T3SS1 inducing and non-inducing conditions

For the LB-S condition (non-inducing) duplicate 16 h cultures of NY-4 were diluted 1:100 into fresh LB-S without antibiotics and incubated at 37°C with shaking for 3 h. For the DMEM condition (inducing) 1 ml of duplicate 16 h cultures of NY-4 were centrifuged and the pellets were resuspended in 10 ml of DMEM supplemented with 1% FBS (v/v) and incubated at 37°C with shaking for 3 h (Zhou et al., 2008). For *in trans exsA* and *exsD* expression (inducing and non-inducing, respectively), duplicate 16 h cultures of NY carrying plasmid pMMB207 containing the *exsA* or *exsD* gene (NY-4:p*exsA*, NY-4:p*exsD*) were diluted 1:1000 into fresh LB-S containing chloramphenicol (8.5 μg ml^−1^) to ensure plasmid retention and incubated at 37°C with shaking to an OD_600_ of 0.3–0.5. Expression of *exsA* and *exsD* was initiated by the addition of IPTG to a final concentration of 1 mM followed by incubation for 3 additional hours.

### HeLa cell infection in hank's balanced salt solution (HBSS) and cytotoxicity assay

Growth media was removed from ~80–100% confluent HeLa cell monolayers, the cells were washed with sterile PBS pre-warmed to 37°C, and the media was replaced with HBSS pre-warmed to 37°C (with calcium, magnesium, and phenol red, Thermo Scientific, Catalog #SH30030.02) supplemented with 1% FBS (v/v). A 16 h culture of NY-4 was diluted 1:50 into fresh LB-S without antibiotics and incubated at 37°C with shaking for 1.5 h, centrifuged and the pellet resuspended in an equivalent volume of HBSS/1% FBS (0 h, pre-infection) before infecting HeLa cells at a multiplicity of infection (m.o.i.) of ~10–20. Bacterial CFU counts were verified using the 6 × 6 drop plate method as previously described (Chen et al., 2003). At the onset of infection cells were centrifuged at 250 g for 4 min to synchronize cell-cell contact and incubated at 37°C with 5% CO_2_. At specified time points (2, 3, 4, 6, 8 h post-infection) 50 μl aliquots of the supernatant were removed to measure lactate dehydrogenase (LDH) release using a CytoTox 96 Non-Radioactive Cytotoxicity Assay (Promega, Catalog #G1780) according to manufacturer's instructions. Supernatant and cells from triplicate wells were removed with a sterile cell scraper along with pipetting and then pooled for RNA extraction. Infection was performed in this manner on two separate occasions to generate independent biological replicates. Maximum LDH release was achieved using provided 10× Lysis Solution and spontaneous LDH release was measured from uninfected HeLa cells. LDH release was obtained by measuring absorbance at 490 nm using a SpectraMax Plus^384^ Absorbance Microplate Reader (Molecular Devices). Percent cytotoxicity was calculated as follows:

$$\%\text{ Cytotoxicity =}\frac{\text{Test LDH release}-\text{Spontaneous release}}{\text{Maximum release}-\text{Spontaneous release}}\times 100$$

### RNA extraction, analysis, and mRNA purification

A total of 3 ml of each sample was withdrawn and total RNA was isolated using a RiboPure-Bacteria kit (Ambion, Catalog #AM1925) with the addition of a second treatment of DNase using a TURBO DNA-free kit (Ambion, Catalog #AM1907) according to manufacturer's instructions. Following extraction RNA was ethanol precipitated as necessary using a modified version of a previously described protocol (Green and Sambrook, 2012). Briefly, 1/10 the volume of 3 M sodium acetate, 2.5 volumes of 100% ethanol and 2 μl GlycoBlue (Ambion, Catalog #AM9515) were added to each sample. Samples were centrifuged at maximum speed and 4°C for 30 min, the supernatant discarded and pellet washed with 200–250 μl of ice-cold 70% ethanol and mixed by inversion. Samples were then centrifuged at maximum speed and 4°C for 15 min, the supernatant discarded and pellet dried. Samples were resuspended with pre-warmed (55°C) DEPC-treated water. RNA samples were quantified using a NanoDrop ND-1000 spectrophotometer and analyzed for quality on an Agilent 2100 Bioanalyzer at Washington State University's Laboratory of Biotechnology and Bioanalysis. Ribosomal RNA was removed using a Ribo-Zero Magnetic Kit (Gram-Negative Bacteria) (Epicentre, Catalog #MRZGN126) according to manufacturer's instructions (Giannoukos et al., 2012).

### Quantitative RT-PCR (qRT-PCR)

Total RNA (100 ng) was used to generate cDNA using an iScript Reverse Transcription Supermix (Bio-Rad, Catalog #170-8841) according to manufacturer's instructions. qPCR was performed in triplicate for each sample using SsoAdvanced SYBR Green Supermix (Bio-Rad, Catalog #172-5261) according to the manufacturer's instructions. Primer pairs used were qExsA-F 5′-tccgtcagcttccactcttt-3′, qExsA-R 5′-ctcgggcttgttttcttttg-3′ and qSecY-F 5′-actggctcagtggtttggtc-3′, qSecY-R 5′-gggtacgaatgcaccagact-3′ (Erwin et al., 2012). Reactions were performed using a CFX C1000 Touch Real-Time PCR Detection System with the following cycling parameters: one cycle of 95°C for 30 s, 38 cycles of 95°C for 1 s, 52°C for 5 s, and 72°C for 15 s. Relative expression levels were calculated using the ΔΔCt method with *secY* as the control transcript (Livak and Schmittgen, 2001; Erwin et al., 2012). Reverse transcriptase negative controls showed either no amplification or Cq values >35 for both *exsA* and *secY* in all samples, indicating negligible levels of DNA contamination.

### RNA sequencing

Purified mRNA was used to prepare individually barcoded (indexed) RNA-Seq libraries with a TruSeq RNA Sample Prep kit (Illumina, Catalog #RS-122-2001). RNA-Seq libraries were sequenced on a MiSeq Benchtop Sequencer (Illumina Inc.) using version 2 chemistry and reads were base called with CASAVA1.8 pipeline (Illumina Inc.) to generate paired-ended 150-bp reads. The data discussed in this publication have been deposited in NCBI's Gene Expression Omnibus (Edgar et al., 2002; Barrett et al., 2009, 2013) and are accessible through GEO Series accession number GSE51423 (http://www.ncbi.nlm.nih.gov/geo/query/acc.cgi?acc=GSE51423). RNA sequence analysis was performed using CLC Genomics Workbench (version 6.0.5). Sequence data was mapped to reference genomes for *V. parahaemolyticus* strain RIMD 2210633 chromosomes 1 and 2 (NCBI Reference Sequence NC_004603.1 and NC_004605.1, respectively) (Makino et al., 2003). Mapping for RNA-Seq was allowed 20 additional upstream and downstream bases for each gene and a paired distance range of 0–300 bases. Up to two mismatches were allowed, with 90% alignment to the reference sequence and 80% similarity required for inclusion as a mapped read.

### Comparative transcriptome analysis

Comparative expression analysis between replicate samples was performed using Baggerly's test and the Bonferroni correction to obtain proportional fold change (Benjamini and Hochberg, 1995; Baggerly et al., 2003; Dudoit et al., 2003); genes that generated infinite fold change on comparative analysis were excluded, as these represented genes with zero mapped reads in their corresponding baseline conditions (DMEM vs. LB-S *n* = 26, ExsA vs. ExsD *n* = 32, 2 h vs. 0 h *n* = 16, 3 h vs. 0 h *n* = 22, 4 h vs. 0 h *n* = 30, 6 h vs. 0 h *n* = 28, 8 h vs. 0 h *n* = 23). Putative functional roles were grouped by Cluster of Orthologous Groups of proteins (COG) designation (Tatusov et al., 1997, 2003) and by association with T3SS1 (*vp1656*—*vp1702*; *vpa0450*—*vpa0451*) or T3SS2 (*vpa1314—vpa1378*).

## Results

### Experimental design

This study was comprised of two major parts: analysis of *V. parahaemolyticus* T3SS1 inducing conditions (growth in DMEM, *in trans exsA* expression) compared to their corresponding non-inducing conditions (growth in LB-S, *in trans exsD* expression) and infection of HeLa cells in HBSS over time compared to pre-infection conditions. Previous experiments demonstrated that growth in DMEM, contact with HeLa cells and *exsA* expression *in trans* induce the T3SS1, while growth in LB-S and *exsD* expression *in trans* do not induce the T3SS1 (Zhou et al., 2008). As media only growth conditions, DMEM and LB-S were considered optimal inducing/non-inducing pairs for analysis, and *in trans exsA* and *exsD* expression were paired because they carried similar plasmid constructs and were treated to identical growth conditions (see Materials and Methods). HBSS served as a minimal media for HeLa cell infection to avoid the T3SS1 inducing effects of DMEM. *V. parahaemolyticus* grows poorly in HBSS due to the relative lack of nutrients, which helped to minimize the growth effects of the media and increase their reliance on the HeLa cells for nutrient acquisition. We surmised that these conditions would yield a less confounded transcriptional profile of *V. parahaemolyticus* cytotoxicity toward HeLa cells. The concentration of FBS was reduced from 10 to 1% for infection to minimize effects on LDH measurement while maintaining HeLa cell viability for the duration of the experiments. Comparative transcriptome profiles were then assembled between T3SS1 inducing and non-inducing conditions, as well as over time during HeLa cell infection and screened for genes that met various upregulation criteria. Genes identified by these criteria were considered genes of interest for further study.

### RNA sequencing and quality control

**Figure 1:**
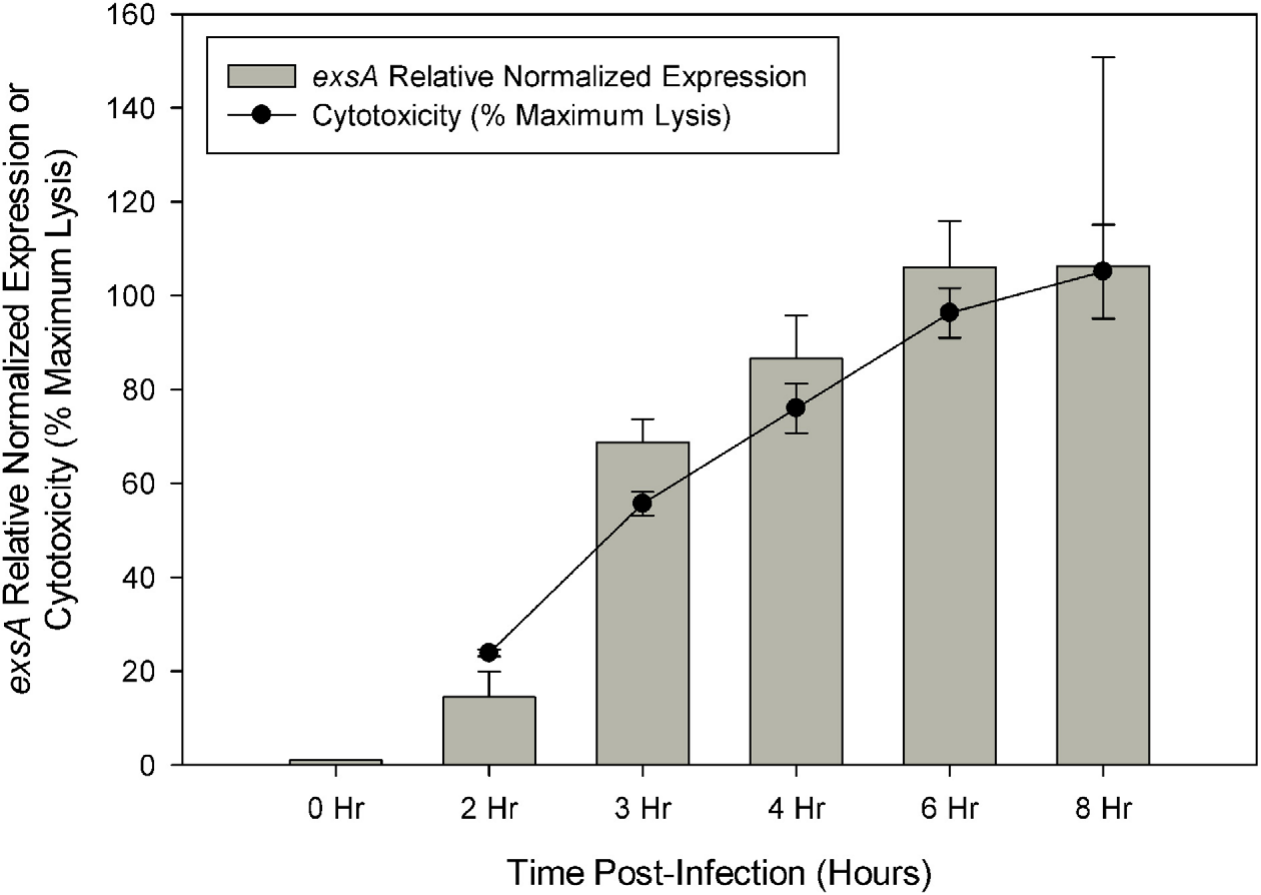
**Quantitive RT-PCR was used to determine the relative expression of *exsA* during HeLa cell infection.** Relative expression at each time point was determined by comparison with 0 h (pre-infection) using the ΔΔCt method with *secY* as the control transcript. Cytotoxicity was assessed by LDH release at specified time points during HeLa cell infection and calculated as a percentage of maximum lysis (see Materials and Methods). Each measurement represents the average of two independent replicates, and error bars represent s.e.m.

Cytotoxicity analysis of *V. parahaemolyticus* strain NY-4 showed that maximal lysis of HeLa cells was reached by approximately 6 h post-infection, which was directly proportional to *exsA* expression as assessed by qRT-PCR (Figure 1). Regarding the RNA-seq data, the total number of reads mapped to the reference genome (in millions) was as follows for each replicate sample: LB-S (10.8, 10.3), DMEM (8.6, 10.1), ExsA (12.5, 11.4), ExsD (13.4, 10.8), 0 h (12.9, 11.8), 2 h (3.84, 4.49), 3 h (3.18, 5), 4 h (5.45, 3.05), 6 h (3.43, 5.78), 8 h (4.27, 3.44). All of the mapped reads were obtained as paired-end reads. Previous studies indicated that a sequencing depth of 2–3 million reads is sufficient to detect genes differentially expressed by 2-fold or more when well correlated biological replicates are present (Haas et al., 2012). The data obtained should therefore provide adequate representation of each condition's transcriptional profile and be suitable for comparative analysis to reliably detect genes with a ≥5-fold change.

**Figure 2:**
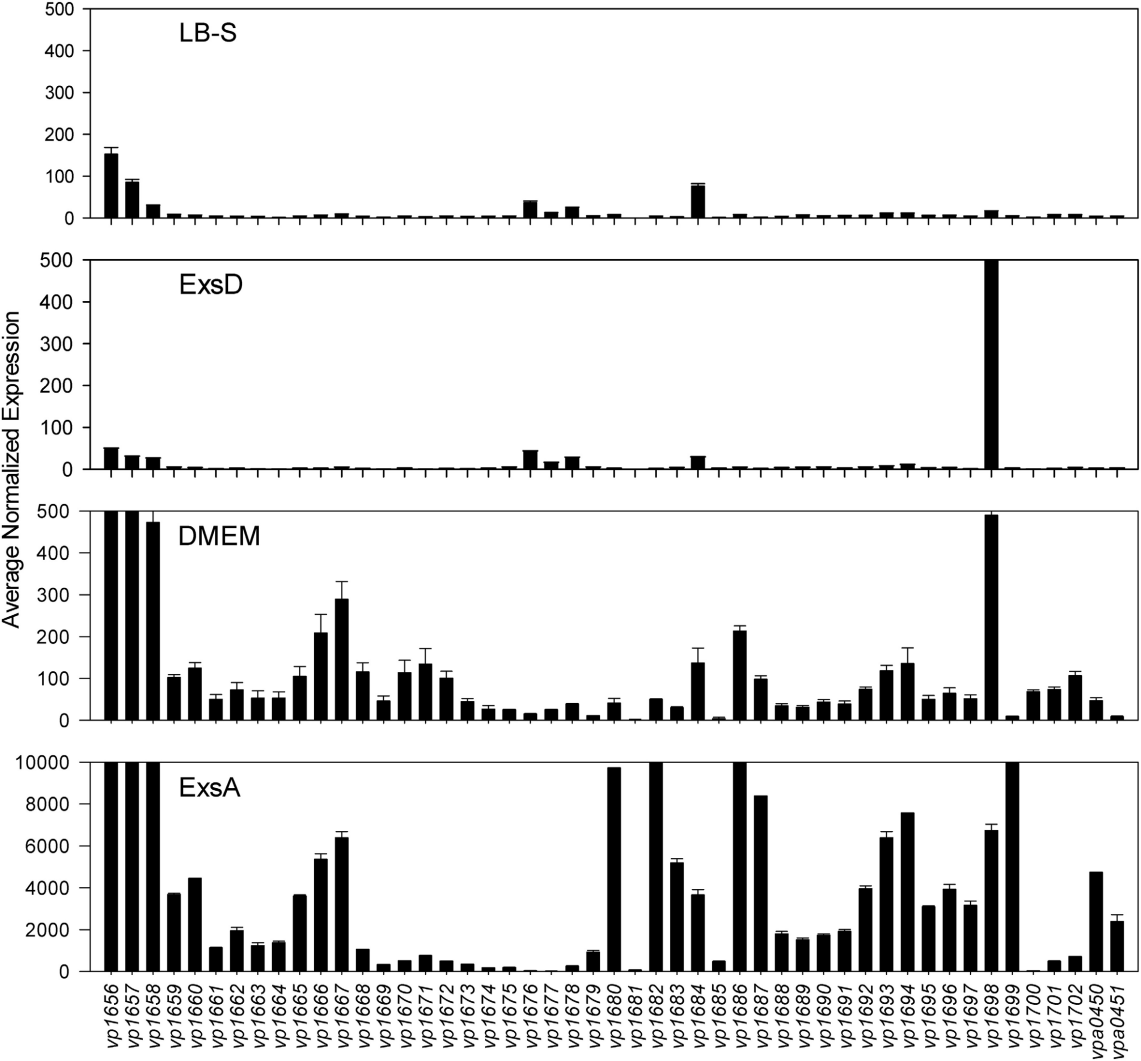
**Average normalized expression (*n* = 2 replicates) for T3SS1 associated genes (*vp1656*—*vp1702*; *vpa0450*—*vpa0451*) in T3SS1 non-inducing (LB-S, ExsD) and inducing (DMEM, ExsA) conditions.** LB-S: NY-4 was subcultured into LB-S media and grown for 3 h. ExsD: NY-4:p*exsD* was subcultured into LB-S and IPTG induced to express *exsD* for 3 h. DMEM: NY-4 was subcultured into DMEM/1% FBS media and grown for 3 h. ExsA: NY-4:p*exsA* was subcultured into LB-S and IPTG induced to express *exsA* for 3 h. (See Materials and Methods for additional detail.) The range of the y-axis for the ExsA condition was increased so expression levels could be viewed. Error bars represent s.e.m.

**Figure 3:**
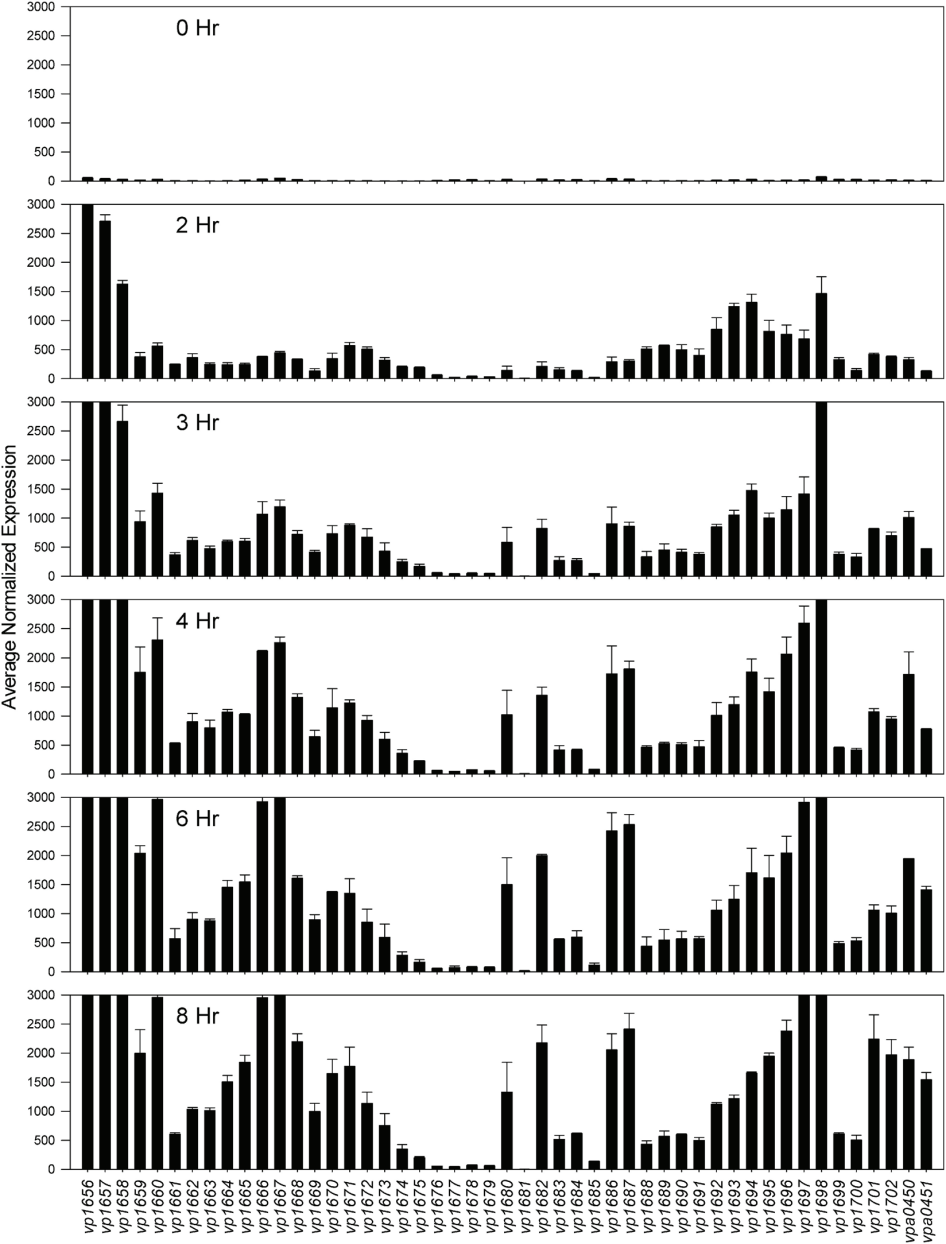
**Average normalized expression (*n* = 2 replicates) for T3SS1 associated genes (*vp1656*—*vp1702*; *vpa0450*—*vpa0451*) at various time points during HeLa cell infection in HBSS/1% FBS with *V. parahaemolyticus* strain NY-4 (m.o.i. ~10–20).** (See Materials and Methods for additional detail.) Error bars represent s.e.m.

Comparative transcriptome analysis was initiated by quantile normalization of all samples to obtain normalized expression values, from which the mean and standard error of the two replicates for each sample were calculated. For the inducing conditions (DMEM media and *in trans exsA* expression), T3SS1 associated genes (*vp1656*—*vp1702*; *vpa0450*—*vpa0451*) were generally upregulated as expected in comparison to the non-inducing conditions [LB-S and *in trans exsD* expression; except for *exsD* (*vp1698*) itself] (Figure 2, Supplementary Table 1). Notably, genes for hydrophobic translocators *vp1656* and *vp1657*, translocator chaperone *vp1658* and CesT family chaperone *vp1684* showed apparent basal expression in non-inducing conditions that increased during inducing conditions. Genes of unknown function *vp1676*—*vp1677* were basally expressed in the non-inducing conditions but not substantially upregulated during inducing conditions, while hypothetical protein gene *vp1681* showed low expression under both non-inducing and inducing conditions. Overexpression of *exsA* also yielded much higher expression levels of genes associated with T3SS1 than DMEM induction, which is expected given the high levels of the T3SS1 transcriptional activator *exsA* (*vp1699*) being produced in this condition. RNA expression analysis during HeLa cell infection showed little to no expression of T3SS1 associated genes at 0 h and increased expression at 2 h that continues through 8 h post-infection (Figure 3, Supplementary Table 2). Genes of unknown function *vp1676—vp1679* and *vp1681* appeared to be minimally upregulated during HeLa cell infection.

### Comparative transcriptome analysis—inducing vs. non-inducing conditions

**Figure 4:**
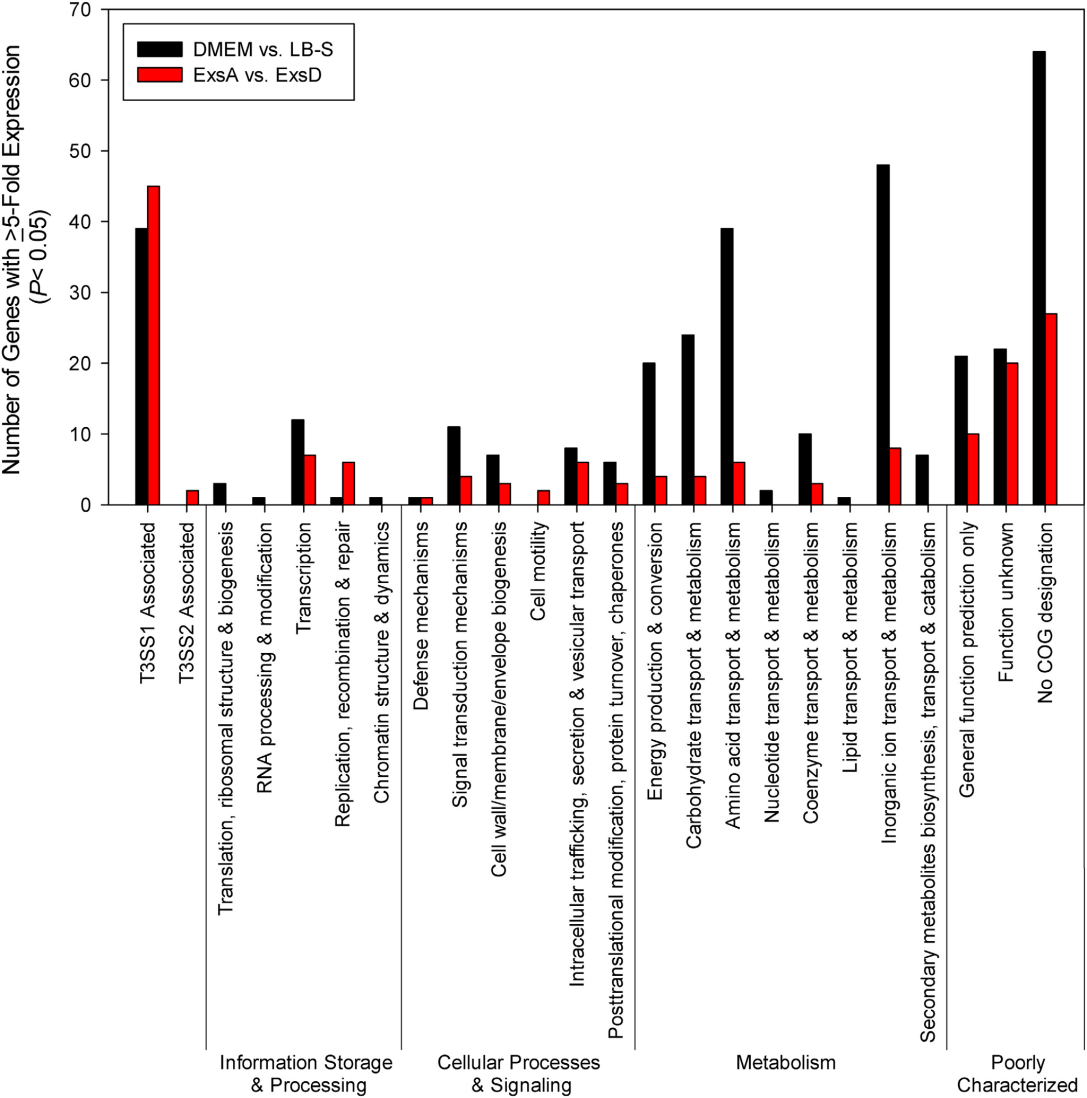
**Induction of T3SS1 in DMEM compared to LB-S and *in trans exsA* expression compared to *in trans exsD* expression (*n* = 2 replicates).** Genes were screened for ≥5-fold increase in expression (*P* < 0.05) and grouped by COG designation and association with T3SS1 (*vp1656*—*vp1702*; *vpa0450*—*vpa0451*) or T3SS2 (*vpa1314—vpa1378*).

Comparison of growth in DMEM to LB-S yielded 332 genes that met upregulation criteria (≥5-fold change, *P* < 0.05), with the majority of upregulated genes associated with metabolism, followed by poorly characterized genes and then T3SS1 associated genes (Figure 4, Supplementary Table 3). Comparison of *in trans exsA* expression to *exsD* expression identified 152 genes meeting these criteria, and in contrast the majority of these genes were associated with T3SS1 or are poorly characterized, with metabolism comprising only a small fraction of the upregulated genes (Figure 4, Supplementary Table 4). In addition, the *exsA* vs. *exsD* comparison identified two upregulated genes associated with T3SS2; hypothetical proteins encoded by *vpa1343* and *vpa1356*. Notable differences were observed in the overall expression patterns between the DMEM vs. LB-S and ExsA vs. ExsD comparative groups. DMEM induction appeared substantially more metabolically active and showed more upregulated genes associated with energy production and conversion and the transport and metabolism of metabolic materials including carbohydrates, amino acids, inorganic ions, and coenzymes as compared to ExsA induction. In addition, DMEM induction showed slightly more genes associated with processes related to information storage and processing and cellular processes and signaling, although comparative *exsA* expression showed slightly more genes associated with replication, recombination and repair and cell motility. The most highly upregulated genes in the comparison between DMEM and LB-S included five genes putatively involved in iron acquisition (TonB proteins *vpa0424* and *vpa0425*, HutB protein *vpa0423*, iron-regulated protein *vpa0664*, and vibrioferrin receptor *vpa1656*) with most of the remaining genes being associated with metabolism (Supplementary Table 3). The most highly upregulated genes in the comparative *exsA*/*exsD* conditions were associated with T3SS1 (Supplementary Table 4). Excluding T3SS1 associated genes there were 13 genes that met upregulation criteria shared between the DMEM/LB-S and *in trans exsA*/*exsD* comparisons, with six genes putatively associated with metabolic processes, three genes associated with cellular processes and signaling and four poorly characterized genes (Table 1).

**Table 1:** Genes showing ≥5-fold change (*P* < 0.05) in expression in DMEM compared to LB-S conditions and *in trans exsA* compared to *in trans exsD* expression^*^.

**Locus tag**	**Putative product**	**COG**
*vp1480*	Riboflavin synthase subunit alpha	COG0307H
*vp2420*	Pilus assembly protein	COG3745U
*vp2422*	Hypothetical protein	COG4960OU
*vp2423*	Fimbrial protein	COG3847U
*vp2596*	LysE/ArgO (YggA) family protein	COG1279R
*vp2597*	DNA-binding protein	COG2606S
*vp2758*	Acetylglutamate kinase	COG0548E
*vpa0159*	Hypothetical protein	COG5266P
*vpa0289*	Hypothetical protein	COG1280E
*vpa0597*	Hypothetical protein	–
*vpa0635*	Oxidoreductase, oxygen dependent, FAD-dependent protein	COG0277C
*vpa0882*	Heme transport protein HutA	COG1629P
*vpa1275*	Short chain dehydrogenase/reductase family oxidoreductase	COG4221R

* T3SS1 associated genes (vp1656—vp1702; vpa0450—vpa0451) are excluded from this table.

### HeLa cell infection—T3SS1 associated genes

**Figure 5:**
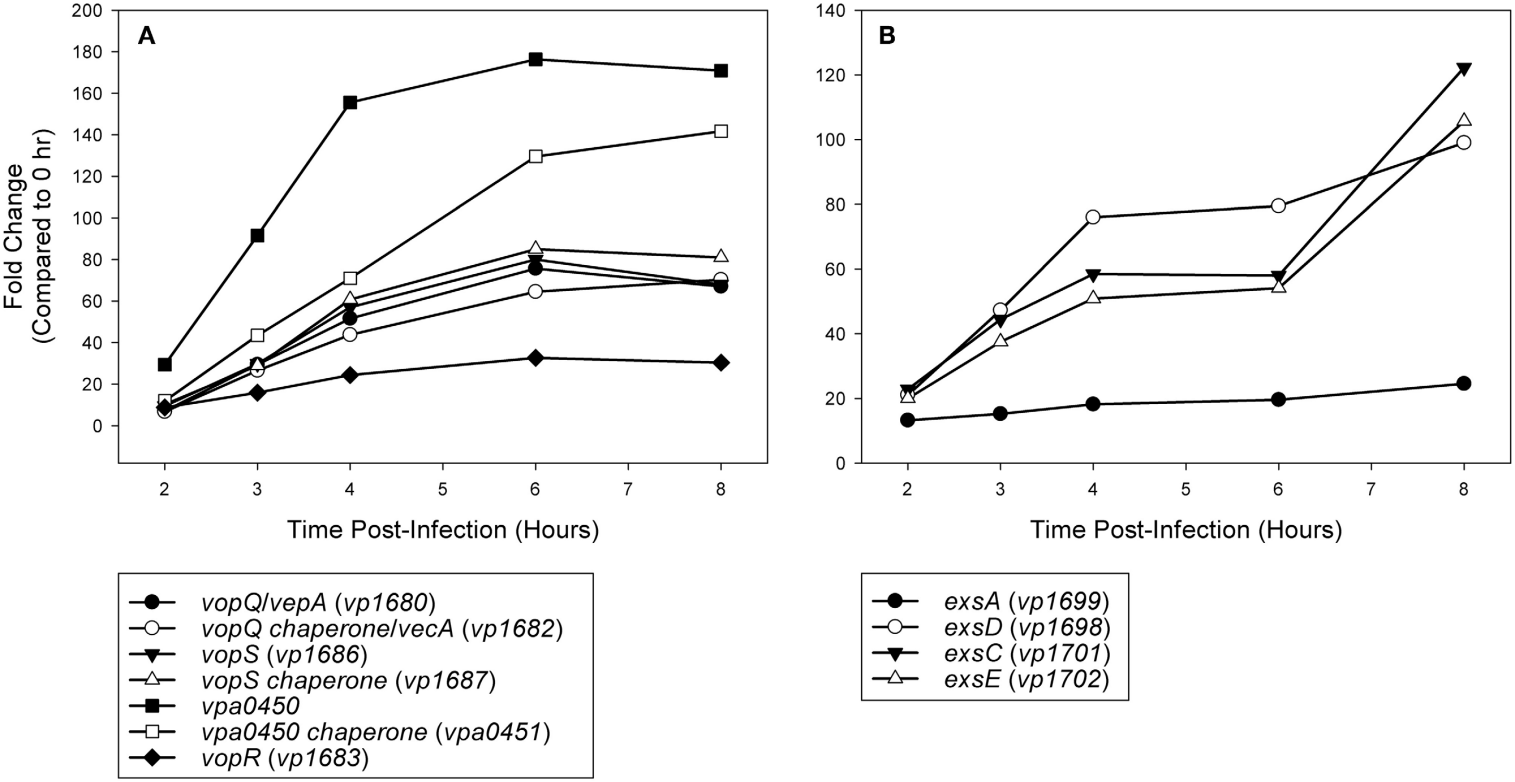
**(A)** Time points during infection of HeLa cells (*n* = 2 replicates) were compared to 0 h (pre-infection) and fold change of known and putative T3SS1 effector protein and effector chaperone genes were plotted over time. **(B)** Time points during infection of HeLa cells (*n* = 2 replicates) were compared to 0 h (pre-infection) and fold change of known T3SS1 regulatory genes were plotted over time.

Expression analysis of replicate samples at each HeLa infection time point were compared to the 0 h (pre-infection) time point using Baggerly's test to obtain proportional fold change. Genes encoding known and putative T3SS1 effector proteins and their cognate chaperones all showed substantial increases in expression during HeLa infection (Figure 5A, Supplementary Table 2). The effector proteins' fold change peaked at 6 h and then began to decline, which coincides with the point at which maximal cytotoxicity was reached, while expression for most of the cognate chaperones continued to increase through 8 h. T3SS1 translocator genes and their chaperones also showed increased expression during infection, with the two hydrophobic translocators *vp1656* and *vp1657* showing the highest expression, primarily at 8 h, which follows the point of maximal cytotoxicity (Supplementary Table 2). Interestingly, there was only a relatively small fold-increase in the expression of the T3SS1 transcriptional regulator gene *exsA* during infection, while T3SS1 anti-activator gene *exsD* showed large increases throughout infection (Figure 5B, Supplementary Table 2). While the magnitude of fold change for *exsA* was approximately 300% less on average for RNA-seq than quantitative PCR (Figure 1), the pattern of change in expression was highly correlated (*r* = 0.85). Expression of the other T3SS1 regulators *exsC* and *exsE* showed substantial increases over the course of infection, culminating in a sharp peak at 8 h (Figure 5B). Most of the T3SS1 structural genes also showed increased expression, including the ruler (*vp1670*), inner rod (*vp1691*), the inner membrane export apparatus (*vp1662*, *vp1672*, *vp1673*, *vp1674*, *vp1675*), ATPase (*vp1668*), cytoplasmic ring (*vp1671*), membrane-supramembrane ring (*vp1690*, *vp1695*), and the outer membrane secretin ring (*vp1696*). In addition, *vp1667*, *vp1665*, *vp1697*, and *vp1666*, which are homologs to YopN, SycN, YscB, and TyeA respectively, showed upregulation during infection (Supplementary Table 2). In *Yersinia* these four proteins form a complex that blocks Yop secretion under non-secretion conditions, and this complex is presumably present in many if not all T3SSs (Galán and Wolf-Watz, 2006).

### Comparative transcriptome analysis—HeLa cell infection

**Figure 6:**
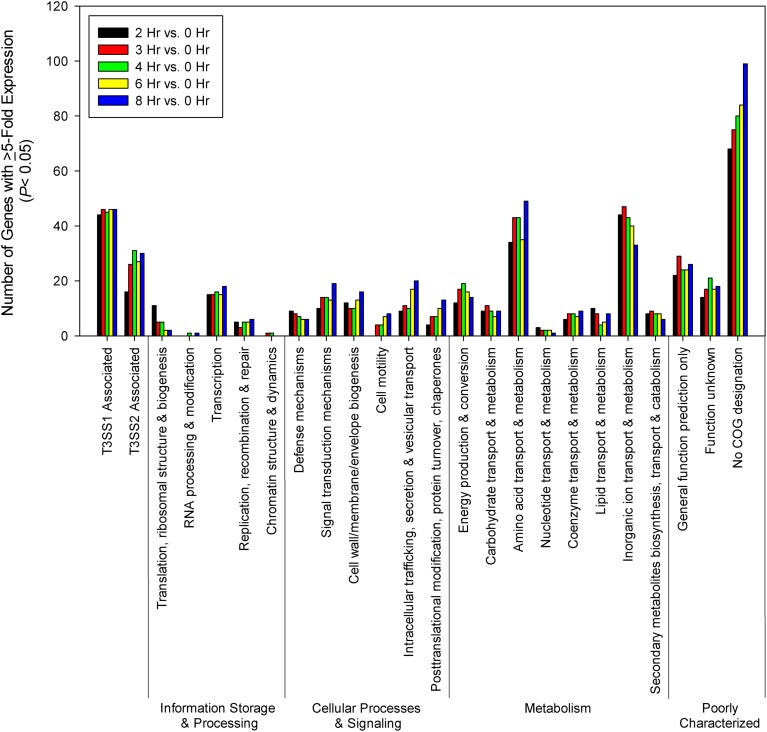
**Time points during infection of HeLa cells (*n* = 2 replicates) were compared to 0 h (pre-infection).** Genes were screened for ≥5-fold increase in expression (*P* < 0.05) and grouped by COG designation and association with T3SS1 (*vp1656*—*vp1702*; *vpa0450*—*vpa0451*) or T3SS2 (*vpa1314—vpa1378*).

For most of the COG associated categories the majority of the genes meeting upregulation criteria (≥5-fold change, *P* < 0.05) were seen by 2 h post-infection, with a relative minority of additional genes meeting upregulation criteria over the remaining course of infection (Figure 6). Given that T3SS1 has been shown to predominate during HeLa cell infection, it was surprising to find that a significant number of genes associated with T3SS2 (*vpa1314—vpa1378*) met upregulation criteria and that several demonstrated continued upregulation throughout the course of HeLa cell infection. The number of transcription-related genes was largely static while those genes associated with translation decreased during infection. The number of genes associated with cellular processes and signaling generally increased during infection with the exception of those involved in defense mechanisms. The number of genes associated with metabolism generally peaked and then declined over the course of infection, with the genes involved in the transport and metabolism of inorganic ions peaking early in infection while those associated with amino acid transport and metabolism appeared to peak at mid-infection and then again late in infection (Figure 6). Similarly, at 2 h post-infection many of the highly upregulated genes (≥150-fold change, *P* < 0.05) were associated with iron acquisition (Supplementary Table 5), but by 3 h post-infection several highly upregulated genes (≥150-fold change, *P* < 0.05) were associated with nitrate and amino acid transport, a trend that persisted through 8 h post-infection (Supplementary Table 6). Excluding T3SS1 associated genes there were 33 genes that showed consistent upregulation and at least a 10-fold increase in expression over the course of HeLa cell infection. Of these 11 genes were associated with metabolism, six were associated with information storage and processing, four were associated with cellular processes and signaling, and 14 were poorly characterized (Table 2). In addition to the upregulated genes, those that were downregulated and static over the course of HeLa cell infection are provided in Supplementary Table 7.

**Table 2:** Genes showing upregulation over the course of HeLa cell infection compared to 0 h (pre-infection), defined as increasing fold change from 3–6 h post-infection, ≥10-fold increase between 2 and 8 h post-infection, and 8 h fold change >0 (*P* < 0.05)^*^.

**Locus tag**	**Putative product**	**COG**	**2 h fold change**	**3 h fold change**	**4 h fold change**	**6 h fold change**	**8 h fold change**
*vp0018*	16 kDa heat shock protein A	COG0071O	1.7	2.5	4.6	15.3	12.6
*vp0481*	Glutamate synthase subunit beta	COG0493ER	7.6	14.3	18.0	20.7	32.4
*vp0483*	Glutamate synthase subunit beta	COG0493ER	44.4	48.8	69.4	88.9	130.2
*vp0985*	SpoVR family protein	COG2719S	3.1	9.8	12.9	18.0	32.1
*vp0986*	Hypothetical protein	COG2718S	2.1	11.3	15.3	25.5	40.7
*vp0987*	Hypothetical protein	–	2.4	9.4	12.7	25.1	29.1
*vp1012*	Cold shock-like protein CspD	COG1278K	7.3	21.6	31.9	42.5	70.0
*vp1205*	Hypothetical protein	–	1.9	3.9	5.2	9.3	13.9
*vp1379*	Homoserine/homoserine lactone efflux protein	COG1280E	3.8	12.8	15.1	19.1	22.1
*vp1605*	Hypothetical protein	–	3.9	4.0	7.8	19.2	39.2
*vp2070*	Hypothetical protein	–	8.6	62.9	70.2	87.1	246.1
*vp2371*	N-acetylglutamate synthase	COG0548E	5.6	8.2	8.7	16.5	16.4
*vp2553*	RNA polymerase sigma factor RpoS	COG0568K	−1.6	2.1	3.1	5.2	10.5
*vp2756*	Bifunctional argininosuccinate lyase/N-acetylglutamate synthase	COG0165E	7.5	13.1	14.4	23.4	24.6
*vp2757*	Argininosuccinate synthase	COG0137E	12.4	29.2	33.1	52.5	65.4
*vp2758*	Acetylglutamate kinase	COG0548E	9.5	21.3	22.6	43.7	45.7
*vp2910*	Hypothetical protein	COG4974L	2.0	4.8	6.7	11.6	15.1
*vpa0035*	Sodium/glutamate symporter	COG0786E	−7.0	1.3	1.7	3.0	3.5
*vpa0639*	Arginine transporter permease subunit ArtM	COG4160E	40.5	90.1	113.9	124.2	57.8
*vpa0669*	Hypothetical protein	–	2.0	4.1	6.2	10.1	12.4
*vpa0680*	Arylsulfatase	COG3119P	6.9	18.4	25.9	40.5	59.9
*vpa0694*	Hypothetical protein	COG3111S	2.1	3.5	5.6	14.7	14.2
*vpa1050*	Hypothetical protein	–	6.0	15.6	20.4	36.7	50.7
*vpa1186*	Outer membrane protein OmpA	COG2885M	4.5	14.3	18.4	19.6	38.2
*vpa1287*	Transporter	COG1230P	2.2	5.9	7.3	12.2	18.2
*vpa1387*	Hypothetical protein	–	2.8	7.5	11.9	15.3	18.8
*vpa1388*	Hypothetical protein	–	6.2	12.7	18.1	21.3	28.1
*vpa1391*	Hypothetical protein	COG1476K	2.4	5.3	6.8	10.3	17.5
*vpa1394*	Transposition protein	COG1474LO	4.4	10.3	13.2	17.1	28.2
*vpa1395*	Transposase	–	5.7	18.1	25.9	37.5	63.1
*vpa1396*	Hypothetical protein	–	2.3	6.9	11.4	15.9	33.5
*vpa1398*	Hypothetical protein	–	4.0	7.4	10.9	16.6	26.3
*vpa1446*	LuxR family transcriptional regulator	COG2197TK	5.8	12.0	14.5	15.0	18.2

* T3SS1 associated genes (vp1656—vp1702; vpa0450—vpa0451) are excluded from this table.

## Discussion

Current high-throughput sequencing technology has enhanced our ability to obtain detailed transcriptomic profiles to improve our understanding of cellular processes and has provided a means for the discovery of novel genes involved in virulence. Herein we used RNA-seq to investigate the activity of T3SS1 in *V. parahaemolyticus* through DMEM media induction, transcription factor *exsA* activation and HeLa cell infection. Expression of known T3SS1 genes encoding effector proteins VopQ (VepA, *vp1680*), VopS (*vp1686*), VPA0450 (*vpa0450*), and putative effector protein VopR (*vp1683*) share a synchronous rise during HeLa cell infection that peaks at 6 h post-infection and then begins to decline (Figure 5A, Supplementary Table 2). This corresponds well with the point at which maximal cytotoxicity was achieved (Figure 1), in contrast to most of the genes encoding effector protein chaperones and translocators, which showed increased expression at 8 h post-infection. Continued expression analysis beyond 8 h post-infection would help to determine whether these findings were spurious or representative of typical expression trends during HeLa cell infection. Most *V. parahaemolyticus* effector proteins and their cognate chaperones are encoded within the same operons, making different expression profiles between the two difficult to explain; if this is the case, it raises the possibility of embedded promoters controlling the expression of some of these genes. The expression pattern of the T3SS1 regulators was particularly surprising; the relatively small changes in the expression of *exsA* throughout infection were unexpected and are seemingly at odds with the magnitude of *exsA* expression observed using quantitative PCR (Figures 1, 5B, Supplementary Table 2). When coupled with the large increases observed in *exsD* expression during infection, several questions arise regarding how T3SS1 remains active during HeLa cell infection. The increased expression of *exsC and exsE* late in infection was also surprising, and this pattern may indicate that changes in expression of these genes have a role in suppressing T3SS1 activity that has not been fully appreciated to date. Further study using proteomic and biochemical investigations on the stability, rate of turnover and stoichiometry of these proteins may help to elucidate some of the answers to these questions. Additionally, upregulation of the YopN (*vp1667*), SycN (*vp1665*), YscB (*vp1697*), and TyeA (*vp1666*) homologs during HeLa infection implies the functional presence of this complex in the regulation of T3SS1 secretion. Genes *vp1666* (*tyeA*) and *vp1667* (*vopN*) have been studied in *V. parahaemolyticus* and found to effect cytotoxicity toward HT-29 human intestinal epithelial cells, but whether they comprise a YopN/SycN/YscB/TyeA regulatory complex as in *Yersinia* is not yet clear (Sarty et al., 2012).

Analysis of the comparative expression patterns between T3SS1 inducing and non-inducing conditions revealed that bacteria induced in DMEM have substantially more upregulated genes associated with metabolic activity than those with *in trans exsA* expression, and a significant number of these genes are associated with the transport and metabolism of carbohydrates, amino acids, coenzymes, and inorganic ions (Figure 4). The finding of genes with increased motility related expression (*vpa0596* and *vpa0747*) in comparative *exsA* analysis is somewhat surprising because overproduction of ExsA has been shown to repress the expression of lateral flagellar genes and inhibit swarming motility in *V. parahaemolyticus* (Gode-Potratz et al., 2010). These two motility associated genes also share COG designation with signal transduction (*vpa0596*) and intracellular trafficking and secretion (*vpa0747*), which may reflect their functions under these conditions. Additionally, in contrast to Gode-Potratz et al. our analysis of downregulated genes during HeLa infection did not show suppression of lateral flagellar genes (Supplementary Table 7B), which may be attributable to the differences in our experimental models. Ultimately, our data suggests that either growth in DMEM provides more signals or opportunities for metabolic activity, or that ExsA overproduction has the capacity to suppress metabolic functions, perhaps in addition to motility.

The gene expression patterns over the course of HeLa infection indicate that the transport of inorganic ions, particularly involving acquisition of iron, are prioritized early during infection, with amino acid and nitrate transport dominating during mid- to late-infection (Figure 6, Supplementary Tables 5, 6); genes involved in these processes may provide effective targets for therapeutic intervention. However, we cannot discount the possibility that transferrins or other iron-binding proteins present in the FBS provided in the culture media (1%) contribute to the upregulation of iron acquisition genes, or that the HBSS media itself is responsible for some of the observed transcriptional changes. Of particular note were the genes associated with T3SS2 that were upregulated (≥5-fold change, *P* < 0.05) during *exsA* expression (*vpa1343, vpa1356*) and during HeLa infection (Figures 4, 6, Supplementary Tables 4, 7C, and data not shown). At 4–6 h, which represented the points of peak cytotoxicity (Figure 1), these included the known T3SS2 effector proteins VopV (*vpa1357*), VopZ (*vpa1336*), and VopL (*vpa1370*) (data not shown). The latter two were also upregulated at 8 h along with hydrophobic translocator VopB2 (*vpa1362*) (data not shown). VopV possesses F-actin-binding activity (Hiyoshi et al., 2011) while VopZ prevents activation of MAPK and NF-κB pathways through inhibition of TAK1 activation (Zhou et al., 2013), and VopL promotes the assembly of actin stress fibers (Liverman et al., 2007). The expression levels of these genes do not fit the pattern of T3SS1 effector protein genes in either intensity or duration, but the possibility that they are exerting an as yet uncharacterized effect during infection cannot be discounted. There were also 9 T3SS2 associated genes that met upregulation criteria at every time point during HeLa cell infection: hypothetical proteins *vpa1324*, *vpa1360*, *vpa1364*, and *vpa1368*, type III secretion proteins *vpa1341*, *vpa1342*, and *vpa1354*, *traA* relaxase *vpa1329* and a T3SS effector and immunogenic protein OspC2 (*vpa1331*) (data not shown). Whether they contribute to HeLa cytotoxicity is also currently unknown.

Through comparative transcriptome analysis several genes have been identified that show upregulation concordant with T3SS1 activation and may play functional roles in *V. parahaemolyticus* virulence. There were 13 unique genes that satisfied upregulation criteria shared between the comparative T3SS1 inducing conditions DMEM and *in trans exsA* expression (Table 1). T3SS1 associated genes were very highly upregulated during *in trans exsA* expression (Supplementary Tables 1, 4), yet the genes identified in Table 1 did not achieve expression levels of this magnitude (Supplementary Tables 3, 4); these genes may reflect differences in the growth conditions or genes relevant to T3SS1 that are not under the direct regulatory control of ExsA. The gene *vp1480* encodes a putative riboflavin synthase subunit; although riboflavin has not frequently been associated with bacterial virulence it has been observed that components of the riboflavin synthesis pathway are essential for *Brucella abortus* survival in cells and mice (Bonomi et al., 2010). Also among the metabolism genes is an acetylglutamate kinase (*vp2758*), an enzyme component involved in the biosynthesis of arginine (Cunin et al., 1986; Xu et al., 2006). Production of arginine may be linked with the LysE/ArgO (YggA) family protein encoded by *vp2596*, which is thought to mediate the export of lysine and/or arginine, respectively. Studies in *E. coli* have demonstrated that lysine and arginine affect *argO* expression, potentially as means of maintaining an optimal ratio of intracellular arginine to lysine (Laishram and Gowrishankar, 2007). Further, analysis of hypothetical protein gene *vpa0289* using the Kyoto Encyclopedia of Genes and Genomes (KEGG) (Kanehisa and Goto, 2000; Kanehisa et al., 2012) indicates it is orthologous to lysine transport genes in several different organisms, which may represent the other functional half of maintaining balance between these amino acids. Iron is an important element for growth, and several bacterial mechanisms have evolved to acquire iron, many of which are associated with bacterial virulence (Litwin and Calderwood, 1993). Upregulation of the gene encoding heme transport protein HutA (*vpa0882*) may be such a candidate. HutA is a heme receptor involved in iron utilization, and a *hutA*-like *V. parahaemolyticus* gene has previously been shown to be functionally interchangeable with the *hutA* gene of *Vibrio cholerae* (O'Malley et al., 1999). Pili (*vp2420*) and fimbrial (*vp2423*) proteins are typically responsible for mediating attachments to cells and other surfaces, although a study in *Pseudomonas aeruginosa* suggested that type 4 pili play a role in cytotoxicity of MDCK cells that is distinct from adherence (Kang et al., 1997). Certain pili have also been associated with biofilm formation in *V. parahaemolyticus* (Yildiz and Visick, 2009). Enzymes like oxidoreductases (*vpa0635*) and dehydrogenases (*vpa1275*) are typically involved in redox reactions important in metabolism and managing oxidative stress (Green and Paget, 2004; White et al., 2012), although there are some studies indicating these enzymes have roles in T3SS function. In *P. aeruginosa*, insertional mutations in the *aceAB* operon, which encodes the PDH-E1 and-E2 subunits of pyruvate dehydrogenase, were unable to activate T3SS genes (Dacheux et al., 2002). In enteropathogenic *E. coli* glyceraldehyde-3-phosphate dehydrogenase is secreted by the T3SS (Aguilera et al., 2012). The function of the genes encoding hypothetical proteins (*vp2422*, *vpa0159*, *vpa0597*) and DNA-binding protein (*vp2597*) identified in this analysis are currently unknown, but KEGG analysis indicates *vp2422* is orthologous to an ABC transporter permease in *Vibrio vulnificus*, and *vpa0159* is orthologous to several nickel transporters. Nickel is an important component in cellular metabolism and as a co-factor for urease, and as such it is important in virulence for bacteria including *Staphylococcus aureus*, *Helicobacter pylori*, and *Brucella* spp. (Roop, 2012; Benoit et al., 2013; Remy et al., 2013).

Comparative transcriptome analysis of *V. parahaemolyticus* during HeLa cell infection yielded 33 unique genes that showed consistent upregulation and a ≥10-fold increase over the course of infection (Table 2). Of these genes only the putative acetylglutamate kinase (*vp2758*) was commonly upregulated during both T3SS1 inducing conditions and HeLa infection (Tables 1, 2). The differences in the transcriptomic profiles implies that there may be distinct repertoires of genes associated with the processes studied here, and in particular that HeLa cell infection induces more than T3SS1 activity. The majority of the metabolism associated genes may have roles in arginine biosynthesis, including the sodium/glutamate symporter (*vpa0035*), glutamate synthase subunits (*vp0481*, *vp0483*), N-acetylglutamate synthase (*vp2371*), arginosuccinate lyase/N-acetylglutamate synthase (*vp2756*), arginosuccinate synthase (*vp2757*), and acetylglutamate kinase (*vp2758*) (Xu et al., 2006). A gene encoding arginine transporter permease subunit ArtM (*vpa0639*) was also upregulated, and has been shown to be a component of a complex that mediates arginine binding and uptake in other organisms (Wissenbach et al., 1993, 1995; Fleischer et al., 2005). Arginine metabolism appears to be associated with HeLa cell infection and cytotoxicity, but transcriptional profiles alone are insufficient to determine the biological role of these pathways. Arginine synthesis and uptake may be for anabolic processes including cell growth and division, but other possibilities include maintenance of an arginine to lysine ratio (Laishram and Gowrishankar, 2007) or use as a component in the export of proteins through a twin-arginine translocation pathway (Palmer and Berks, 2012). Although the latter has not been demonstrated in *V. parahaemolyticus* it does play a role in *Yersinia pseudotuberculosis* virulence when administered orally or intraperitoneally in mice (Lavander et al., 2006). The upregulated putative arylsulfatase (*vpa0680*) may be involved in sulfur acquisition, which is essential for bacterial growth and survival (Kertesz, 2000). An arylsulfatase gene cluster has been described in *V. vulnificus* (Cohen et al., 2007) and sulfatase genes have been suggested to be a component in regulating host-pathogen interactions in mycobacteria (Mougous et al., 2002).

RpoS is an alternative sigma factor that is typically involved in stress responses, but can have a variety of effects on virulence in several species of pathogenic bacteria (Dong and Schellhorn, 2010). Similarly, OmpA has demonstrated a variety of roles including virulence in several pathogens (Confer and Ayalew, 2013). The *rpoS* gene is implicated in survival under hyperosmolar and cold stress conditions in *V. parahaemolyticus* (Vasudevan and Venkitanarayanan, 2006) as well as a variety of stress responses, virulence toward zebrafish and outer membrane protein production in *V. alginolyticus* (Shuxian et al., 2012). This may connect the *rpoS* gene identified (*vp2553*) with the upregulated outer membrane protein gene *vpa1186*.

LuxR is the master regulator of quorum sensing in the related organism *Vibrio harveyi*; its ortholog in *V. parahaemolyticus* is OpaR (*vp2516*), which has been shown to be repressive toward T3SS1 (Gode-Potratz and McCarter, 2011; Zhang et al., 2012). Acyl-homoserine lactones are utilized as chemical signals important in quorum sensing in many proteobacteria, including *Vibrio fischeri* and *V. harveyi* (Pappas et al., 2004). It is currently unknown whether the LuxR family transcriptional regulator (*vpa1446*) and homoserine lactone efflux protein (*vp1379*) upregulated during HeLa cell infection play similar roles in relation to T3SS1 activation and/or quorum sensing in *V. parahaemolyticus*. Upregulation of a heat shock protein (*vp0018*) was detected during HeLa cell infection; a similar protein in *V. harveyi* binds aggregated proteins during heat shock (Klein et al., 2001). Upregulation of a gene encoding cold shock-like protein CspD (*vp1012*) was also detected. Cold-shock proteins can regulate a wide range of cellular functions in bacteria, including virulence factors; in *Bordetella bronchiseptica* the *cspC* and *cspD* genes appear to be repressed by the virulence regulatory BvgAS two-component system, while in *Listeria monocytogenes* Csps have been found to regulate hemolysis and listeriolysin production (Stübs et al., 2005; Schärer et al., 2013). To the author's knowledge, the role of Csps in *V. parahaemolyticus* virulence has not been investigated to date. Transposition proteins (*vpa1394*) and transposases (*vpa1395*) are often associated with mobile genetic elements, and can have roles in transfer of traits encoding antibiotic resistance, mutation, environmental adaptation, and horizontal gene transfer (Casacuberta and González, 2013), but whether they have a connection with T3SS1 is unclear at present.

Several genes encoding hypothetical proteins (*vp0986*, *vp0987*, *vp1205*, *vp1605*, *vp2070*, *vp2910*, *vpa0669*, *vpa0694*, *vpa1050*, *vpa1387*, *vpa1388*, *vpa1391*, *vpa1396*, *vpa1398*), a SpoVR family protein (*vp0985*) associated with *Bacillus* spore formation (Beall and Moran, 1994) and a presumptive cation transporter (*vpa1287*) were also identified and have unknown functions. KEGG analysis has indicated that *vp2910* is orthologous to several integrase/recombinase family proteins, *vpa0694* is orthologous to periplasmic OB (BOF) protein YgiW in *V. fischeri*, *vpa1391* is orthologous to a xenobiotic response element (XRE) family transcriptional regulator and *vpa1396* is orthologous to a TnsA endonuclease, a component of Tn7 transposition. The *vpa1388* gene is orthologous to genes encoding Clustered Regularly Interspaced Short Palindromic Repeats (CRISPR) associated proteins in several different organisms. CRISPRs function as form of RNA-based adaptive immunity systems in bacteria and archaea that mediate the destruction of foreign DNA, but whether they have a role in T3SS1 related virulence is currently unknown (Wiedenheft et al., 2012).

In this study we used RNA-seq to assess the comparative transcriptional profiles of *V. parahaemolyticus* under various T3SS1 inducing conditions and during HeLa cell infection at a level of detail that would not have been possible prior to modern sequencing technology. In doing so, we have been able to observe the general expression changes occurring under each of these conditions as well as identify several genes of interest that may be associated with T3SS1 activity and are worthy of further study. Although RNA-seq is a powerful technique for screening expression profiles, it frequently generates more questions than answers, and it is important to remain aware of its limitations. Gene expression does not necessarily amount to translation into protein, and genes encoding highly labile proteins may be expressed more frequently and at higher levels. In addition, the magnitude of fold change does not necessarily reveal the relative importance of the traits being expressed, as *exsA* showed only modest upregulation whilst it is requisite for expression of T3SS1 (Zhou et al., 2008). Further, transcriptome analysis often cannot delineate protein-protein interactions, particularly between existing or stored pools of material. Additional study of transcriptional profiles utilizing gene deletion mutants, coupling transcriptional data with proteomic studies, targeted gene deletion, and biochemical analysis will help to build on the information obtained in this study and further improve our understanding of T3SS related virulence in *V. parahaemolyticus.*

## Author contributions

Seth D. Nydam was responsible for execution of the experiments and data analysis. Seth D. Nydam and Douglas R. Call were responsible for experimental design, and Seth D. Nydam, Douglas R. Call, and Devendra H. Shah were responsible for writing of the manuscript.

### Conflict of interest statement

The authors declare that the research was conducted in the absence of any commercial or financial relationships that could be construed as a potential conflict of interest.

## Abbreviations

COG: Cluster of Orthologous Groups of proteins
CRISPR: Clustered Regularly Interspaced Short Palindromic Repeats
HBSS: Hank's Balanced Salt Solution
DMEM: Dulbecco's Modified Eagle Medium
KEGG: Kyoto Encyclopedia of Genes and Genomes
LB-S: Luria-Bertani medium supplemented with 2.5% (w/v) NaCl
T3SS: Type III Secretion System
*V. parahaemolyticus*: *Vibrio parahaemolyticus*
XRE: Xenobiotic Response Element.
