# Supplementary material for: Transcriptome analysis of Vibrio parahaemolyticus in type III secretion system 1 inducing conditions
Source: Front Cell Infect Microbiol. 2014 Jan 20;4:1. doi: 10.3389/fcimb.2014.00001 (PMC3895804; doi:10.3389/fcimb.2014.00001)
Supplement: Supplementary file 1 [file DataSheet1.DOCX]

Supplementary Table 1. Average normalized expression (n=2 replicates) and standard error values (in parentheses) for T3SS1 associated genes (*vp1656 – vp1702; vpa0450 – vpa0451*) in T3SS1 non-inducing (LB-S, ExsD) and inducing (DMEM, ExsA) conditions (see Material and Methods).

| **Functional Class** | **Identity** | **Locus Tag** | **Putative Function** | **LB-S** | **ExsD** | **DMEM** | **ExsA** |
| --- | --- | --- | --- | --- | --- | --- | --- |
| Effector | VopQ/VepA | *vp1680* | Autophagy effector protein | 6.9  (0.9) | 1.8  (0.4) | 41.3  (11.0) | 9725.5  (0.0) |
|  | VopR | *vp1683* | Unknown - Putative effector protein | 2.3  (0.4) | 4.0  (0.0) | 30.0  (2.1) | 5182.0  (201.2) |
|  | VopS | *vp1686* | Rho GTPase inhibition effector protein, actin rearrangement | 6.0  (2.2) | 4.7  (0.1) | 213.1  (12.6) | 17785.1  (870.4) |
|  | VPA0450 | *vpa0450* | Inositol phosphatase effector protein | 4.0  (0.1) | 2.2  (0.2) | 46.6  (7.3) | 4734.5  (0.0) |
|  |  |  |  |  |  |  |  |
| Chaperone (Effector) | VopQ chaperone/VecA | *vp1682* | VopQ chaperone | 2.4  (2.1) | 1.4  (0.5) | 49.7  (1.4) | 13969.4  (0.0) |
|  | VopS chaperone | *vp1687* | Putative VopS chaperone | 2.0  (0.1) | 1.0  (0.8) | 98.2  (7.8) | 8380.3  (0.0) |
|  | VPA0450 chaperone | *vpa0451* | Putative VPA0450 chaperone | 4.4  (0.2) | 2.6  (0.1) | 8.4  (1.8) | 2383.1  (324.3) |
|  |  |  |  |  |  |  |  |
| Translocator | YopD homolog | *vp1656* | Hydrophobic translocator | 152.6  (16.0) | 47.0  (3.4) | 1850.2  (354.1) | 52973.6  (0.0) |
|  | YopB homolog | *vp1657* | Hydrophobic translocator | 85.3  (6.8) | 29.0  (2.1) | 1227.3  (268.8) | 20891.3  (2235.7) |
|  | LcrV homolog | *vp1659* | Hydrophilic translocator, Injectisome Tip | 8.2  (0.6) | 4.8  (0.5) | 102.3  (6.7) | 3679.5  (57.4) |
|  |  |  |  |  |  |  |  |
| Chaperone (Translocator) | LcrH homolog | *vp1658* | Class II translocator chaperone | 30.7  (0.2) | 23.4  (3.5) | 472.7  (80.7) | 16278.9  (635.9) |
|  | LcrG homolog | *vp1660* | LcrV chaperone, negative regulator of effector secretion | 5.2  (2.1) | 2.3  (1.4) | 124.4  (13.3) | 4447.7  (0.0) |
|  |  |  |  |  |  |  |  |
| Regulator | ExsD | *vp1698* | Negative regulator of T3SS1 activity | 15.7  (1.4) | 52973.6  (0.0) | 489.7  (92.3) | 6733.5  (300.9) |
|  | ExsA | *vp1699* | Positive regulator of T3SS1 activity | 2.6  (2.4) | 2.1  (0.6) | 9.3  (0.7) | 19385.0  (3742.0) |
|  | ExsC | *vp1701* | Putative ExsD inhibitor | 4.9  (3.0) | 1.4  (0.4) | 73.5  (6.0) | 484.1  (24.2) |
|  | ExsE | *vp1702* | Putative ExsC inhibitor | 6.8  (0.9) | 2.6  (1.7) | 106.5  (10.2) | 708.4  (6.0) |
|  |  |  |  |  |  |  |  |
| Regulator/Not determined | LcrR homolog | *vp1661* | Regulator, low calcium response protein | 4.1  (0.5) | 0.7  (0.6) | 50.0  (11.5) | 1129.2  (29.8) |
|  |  |  |  |  |  |  |  |
| Regulator (Structural) | YscP homolog | *vp1670* | Ruler - Needle length control, substrate specificity switch | 4.3  (0.2) | 1.7  (1.2) | 113.5  (29.6) | 511.7  (6.6) |
|  |  |  |  |  |  |  |  |
| Structural | YscV homolog | *vp1662* | Inner Membrane export apparatus | 3.9  (0.2) | 2.1  (0.3) | 72.6  (17.3) | 1950.8  (152.6) |
|  | YscN homolog | *vp1668* | ATPase | 4.2  (0.1) | 1.6  (0.1) | 115.3  (22.0) | 1057.3  (7.6) |
|  | YscQ homolog | *vp1671* | Cytoplasmic ring - Sorting platform for T3S cargo proteins | 3.1  (0.1) | 0.3  (0.0) | 134.1  (36.9) | 758.9  (1.6) |
|  | YscR homolog | *vp1672* | Inner Membrane export apparatus | 3.6  (1.0) | 0.9  (0.7) | 100.4  (16.8) | 488.6  (15.6) |
|  | YscS homolog | *vp1673* | Inner Membrane export apparatus | 3.6  (0.3) | 0.7  (0.6) | 44.7  (7.2) | 342.8  (2.8) |
|  | YscT homolog | *vp1674* | Inner Membrane export apparatus | 3.5  (0.6) | 2.1  (0.3) | 26.9  (7.9) | 178.1  (2.7) |
|  | YscU homolog | *vp1675* | Inner Membrane export apparatus | 4.9  (0.1) | 5.0  (0.2) | 25.1  (1.1) | 190.9  (3.7) |
|  | YscJ homolog | *vp1690* | Membrane and Supramembrane (MS) ring | 5.1  (0.2) | 2.7  (2.5) | 43.5  (5.9) | 1731.7  (66.5) |
|  | YscI homolog | *vp1691* | Inner rod protein | 5.6  (0.2) | 1.5  (1.4) | 38.7  (7.2) | 1910.8  (90.6) |
|  | YscF homolog | *vp1694* | Needle protein | 10.9  (0.8) | 10.5  (1.1) | 135.4  (37.6) | 7576.8  (0.0) |
|  | YscD homolog | *vp1695* | Membrane and Supramembrane (MS) ring | 5.4  (0.8) | 3.5  (0.1) | 50.2  (8.9) | 3088.2  (54.8) |
|  | YscC homolog | *vp1696* | Outer Membrane secretin ring | 6.1  (0.8) | 4.1  (0.1) | 64.1  (13.3) | 3929.8  (236.1) |
|  |  |  |  |  |  |  |  |
| Chaperone (Structural) | YscG homolog | *vp1693* | Class III chaperone | 11.1  (0.9) | 7.7  (0.4) | 118.2  (12.8) | 6392.5  (289.5) |
|  | YscW homolog | *vp1700* | Pilotin lipoprotein | 1.2  (0.9) | 0.3  (0.0) | 68.3  (4.4) | 26.8  (0.3) |
|  |  |  |  |  |  |  |  |
| Not determined | YscY homolog | *vp1663* | Putative YscX chaperone | 2.3  (1.6) | 0.2  (0.2) | 52.9  (17.5) | 1231.5  (138.1) |
|  | YscX homolog | *vp1664* | Unknown | 1.2  (0.1) | 0.1  (0.1) | 52.8  (14.9) | 1372.7  (68.3) |
|  | SycN homolog | *vp1665* | YopN/SycN/YscB/TyeA complex | 4.1  (0.6) | 1.9  (0.2) | 104.8  (23.9) | 3601.5  (56.7) |
|  | TyeA homolog | *vp1666* | YopN/SycN/YscB/TyeA complex | 6.1  (1.2) | 1.2  (1.1) | 208.4  (44.9) | 5364.6  (261.4) |
|  | YopN homolog | *vp1667* | YopN/SycN/YscB/TyeA complex | 8.3  (1.2) | 4.3  (0.5) | 289.0  (42.5) | 6392.5  (289.5) |
|  | YscO homolog | *vp1669* | Unknown | 1.8  (0.1) | 0.1  (0.0) | 45.7  (12.1) | 329.9  (4.5) |
|  |  | *vp1676* | Putative LysR-family transcriptional regulator | 36.6  (4.0) | 41.5  (2.4) | 15.1  (0.7) | 38.9  (3.4) |
|  | Hypothetical protein | *vp1677* | Unknown | 12.8  (0.2) | 14.3  (2.2) | 25.3  (0.7) | 22.1  (0.2) |
|  |  | *vp1678* | Putative dienelactone hydrolase and related enzymes | 23.1  (2.8) | 26.6  (2.1) | 38.9  (1.3) | 251.9  (33.2) |
|  | Hypothetical protein | *vp1679* | Unknown | 5.0  (0.2) | 4.9  (0.2) | 11.1  (0.2) | 924.5  (78.0) |
|  | Hypothetical protein | *vp1681* | Unknown | 0.0  (0.0) | 0.0  (0.0) | 1.1  (1.1) | 60.2  (23.7) |
|  | CesT family chaperone | *vp1684* | Unknown | 76.0  (5.9) | 28.8  (0.6) | 136.9  (35.6) | 3655.2  (252.0) |
|  | Hypothetical protein | *vp1685* | Unknown | 1.1  (0.1) | 1.2  (0.8) | 3.4  (3.4) | 475.8  (22.3) |
|  | YscL homolog | *vp1688* | Interactor of ATPase/C ring | 3.9  (0.0) | 3.9  (0.0) | 34.6  (4.8) | 1793.4  (117.0) |
|  | YscK homolog | *vp1689* | Interactor of ATPase/C ring | 7.0  (0.6) | 3.7  (0.9) | 31.3  (3.8) | 1508.4  (91.0) |
|  | YscH homolog | *vp1692* | Encodes YopR - Unknown function | 6.2  (0.1) | 4.6  (0.7) | 73.9  (5.6) | 3959.1  (131.1) |
|  | YscB homolog | *vp1697* | YopN/SycN/YscB/TyeA complex | 2.8  (1.7) | 1.2  (0.1) | 51.3  (9.2) | 3154.3  (212.0) |
|  |  |  |  |  |  |  |  |
