# Supplementary material for: Transcriptome analysis of Vibrio parahaemolyticus in type III secretion system 1 inducing conditions
Source: Front Cell Infect Microbiol. 2014 Jan 20;4:1. doi: 10.3389/fcimb.2014.00001 (PMC3895804; doi:10.3389/fcimb.2014.00001)
Supplement: Supplementary file 2 [file DataSheet2.DOCX]

Supplementary Table 2. Average normalized expression (n=2 replicates) and standard error values (in parentheses) for T3SS1 associated genes (*vp1656 – vp1702; vpa0450 – vpa0451*) at various time points during HeLa cell infection with *V. parahaemolyticus* strain NY-4 (See Material and Methods).

| **Functional Class** | **Identity** | **Locus Tag** | **Putative Function** | **0 Hr** | **2 Hr** | **3 Hr** | **4 Hr** | **6 Hr** | **8 Hr** |
| --- | --- | --- | --- | --- | --- | --- | --- | --- | --- |
| Effector | VopQ/VepA | *vp1680* | Autophagy effector protein | 19.8 | 140.2 | 583.9 | 1023.1 | 1499.8 | 1329.5 |
|  |  |  |  | (10.8) | (72.0) | (257.8) | (418.0) | (461.9) | (510.7) |
|  | VopR | *vp1683* | Unknown - Putative effector protein | 17.0 | 149.3 | 270.1 | 413.7 | 553.9 | 515.4 |
|  |  |  |  | (3.1) | (37.2) | (66.8) | (76.7) | (12.5) | (68.2) |
|  | VopS | *vp1686* | Rho GTPase inhibition effector protein, actin rearrangement | 30.3 | 287.0 | 901.6 | 1724.2 | 2420.7 | 2054.5 |
|  |  |  |  | (10.9) | (84.2) | (289.5) | (480.1) | (317.2) | (277.7) |
|  | VPA0450 | *vpa0450* | Inositol phosphatase effector protein | 11.0 | 324.0 | 1009.9 | 1715.3 | 1943.6 | 1884.4 |
|  |  |  |  | (3.7) | (39.4) | (103.9) | (388.2) | (0.0) | (219.1) |
|  |  |  |  |  |  |  |  |  |  |
| Chaperone (Effector) | VopQ chaperone/VecA | *vp1682* | VopQ chaperone | 31.0 | 210.1 | 823.1 | 1354.8 | 1997.4 | 2176.4 |
|  |  |  |  | (6.8) | (77.8) | (157.7) | (141.3) | (19.1) | (306.0) |
|  | VopS chaperone | *vp1687* | Putative VopS chaperone | 29.8 | 305.2 | 861.3 | 1809.9 | 2531.6 | 2413.3 |
|  |  |  |  | (4.6) | (24.5) | (68.6) | (133.6) | (175.8) | (269.1) |
|  | VPA0450 chaperone | *vpa0451* | Putative VPA0450 chaperone | 10.9 | 130.0 | 472.5 | 772.3 | 1409.1 | 1541.3 |
|  |  |  |  | (1.9) | (7.7) | (2.8) | (11.8) | (62.0) | (123.9) |
|  |  |  |  |  |  |  |  |  |  |
| Translocator | YopD homolog | *vp1656* | Hydrophobic translocator | 50.5 | 3014.3 | 5864.5 | 11149.0 | 7760.4 | 16312.5 |
|  |  |  |  | (9.6) | (95.2) | (238.5) | (5765.8) | (1965.2) | (2343.1) |
|  | YopB homolog | *vp1657* | Hydrophobic translocator | 32.4 | 2706.9 | 4748.7 | 6611.7 | 6258.6 | 14649.9 |
|  |  |  |  | (8.9) | (111.9) | (354.4) | (1768.5) | (775.9) | (2264.9) |
|  | LcrV homolog | *vp1659* | Hydrophilic translocator, Injectisome Tip | 18.3 | 372.9 | 938.3 | 1753.2 | 2039.9 | 1994.9 |
|  |  |  |  | (1.1) | (74.3) | (187.1) | (433.9) | (129.5) | (408.0) |
|  |  |  |  |  |  |  |  |  |  |
| Chaperone (Translocator) | LcrH homolog | *vp1658* | Class II translocator chaperone | 23.1 | 1626.4 | 2661.0 | 3436.3 | 3827.7 | 3929.9 |
|  |  |  |  | (9.6) | (65.5) | (281.3) | (108.5) | (534.4) | (236.1) |
|  | LcrG homolog | *vp1660* | LcrV chaperone, negative regulator of effector secretion | 28.4 | 558.3 | 1429.4 | 2305.0 | 2965.3 | 2959.0 |
|  |  |  |  | (2.0) | (55.4) | (169.9) | (377.4) | (177.7) | (74.4) |
|  |  |  |  |  |  |  |  |  |  |
| Regulator | ExsD | *vp1698* | Negative regulator of T3SS1 activity | 69.3 | 1460.5 | 3274.6 | 5264.8 | 5504.6 | 6858.2 |
|  |  |  |  | (6.0) | (293.3) | (270.1) | (530.4) | (121.4) | (176.2) |
|  | ExsA | *vp1699* | Positive regulator of T3SS1 activity | 24.9 | 328.9 | 378.5 | 452.4 | 487.3 | 610.1 |
|  |  |  |  | (8.6) | (32.5) | (38.6) | (10.1) | (31.0) | (20.6) |
|  | ExsC | *vp1701* | Putative ExsD inhibitor | 18.3 | 417.7 | 813.6 | 1070.9 | 1061.6 | 2239.1 |
|  |  |  |  | (1.2) | (19.4) | (9.8) | (54.5) | (88.3) | (418.8) |
|  | ExsE | *vp1702* | Putative ExsC inhibitor | 18.6 | 372.7 | 699.2 | 947.7 | 1008.4 | 1969.8 |
|  |  |  |  | (5.0) | (17.9) | (61.4) | (41.7) | (125.2) | (262.5) |
|  |  |  |  |  |  |  |  |  |  |
| Regulator/Not determined | LcrR homolog | *vp1661* | Regulator, low calcium response protein | 4.2 | 247.9 | 369.7 | 531.3 | 567.5 | 603.6 |
|  |  |  |  | (0.3) | (0.5) | (34.7) | (7.3) | (176.1) | (25.1) |
|  |  |  |  |  |  |  |  |  |  |
| Regulator (Structural) | YscP homolog | *vp1670* | Ruler - Needle length control, substrate specificity switch | 7.0 | 343.7 | 730.4 | 1142.6 | 1371.0 | 1649.8 |
|  |  |  |  | (1.8) | (94.7) | (140.9) | (328.6) | (10.2) | (244.1) |
|  |  |  |  |  |  |  |  |  |  |
| Structural | YscV homolog | *vp1662* | Inner Membrane export apparatus | 5.7 | 361.3 | 614.1 | 900.9 | 902.7 | 1033.7 |
|  |  |  |  | (1.5) | (66.8) | (51.4) | (143.6) | (113.7) | (31.2) |
|  | YscN homolog | *vp1668* | ATPase | 24.7 | 331.8 | 718.8 | 1324.0 | 1609.9 | 2195.5 |
|  |  |  |  | (1.8) | (8.9) | (65.4) | (57.1) | (38.4) | (136.7) |
|  | YscQ homolog | *vp1671* | Cytoplasmic ring - Sorting platform for T3S cargo proteins | 6.1 | 568.1 | 874.6 | 1222.5 | 1346.3 | 1772.3 |
|  |  |  |  | (0.2) | (53.3) | (23.8) | (55.4) | (253.0) | (331.2) |
|  | YscR homolog | *vp1672* | Inner Membrane export apparatus | 5.0 | 506.5 | 673.5 | 924.0 | 853.2 | 1134.6 |
|  |  |  |  | (0.4) | (36.8) | (140.6) | (85.7) | (224.5) | (192.5) |
|  | YscS homolog | *vp1673* | Inner Membrane export apparatus | 2.5 | 318.4 | 431.6 | 602.0 | 588.4 | 755.2 |
|  |  |  |  | (0.5) | (43.0) | (140.7) | (116.1) | (230.4) | (203.2) |
|  | YscT homolog | *vp1674* | Inner Membrane export apparatus | 1.7 | 203.4 | 250.0 | 359.6 | 285.5 | 347.1 |
|  |  |  |  | (0.6) | (11.1) | (39.2) | (59.5) | (58.4) | (80.2) |
|  | YscU homolog | *vp1675* | Inner Membrane export apparatus | 2.5 | 188.6 | 166.2 | 227.4 | 165.1 | 199.4 |
|  |  |  |  | (0.3) | (9.4) | (41.7) | (2.6) | (41.6) | (20.7) |
|  | YscJ homolog | *vp1690* | Membrane and Supramembrane (MS) ring | 9.4 | 496.5 | 410.6 | 509.1 | 565.6 | 597.3 |
|  |  |  |  | (0.5) | (87.1) | (51.9) | (29.5) | (130.6) | (10.8) |
|  | YscI homolog | *vp1691* | Inner rod protein | 6.4 | 400.4 | 379.7 | 471.1 | 564.9 | 495.7 |
|  |  |  |  | (1.1) | (110.0) | (26.8) | (108.5) | (38.1) | (50.8) |
|  | YscF homolog | *vp1694* | Needle protein | 25.2 | 1313.3 | 1473.9 | 1756.6 | 1705.2 | 1655.1 |
|  |  |  |  | (4.2) | (140.5) | (113.1) | (221.7) | (419.6) | (21.2) |
|  | YscD homolog | *vp1695* | Membrane and Supramembrane (MS) ring | 9.1 | 814.1 | 1003.5 | 1419.7 | 1612.6 | 1947.7 |
|  |  |  |  | (2.4) | (188.4) | (83.4) | (228.6) | (388.8) | (53.7) |
|  | YscC homolog | *vp1696* | Outer Membrane secretin ring | 14.3 | 760.6 | 1142.4 | 2066.3 | 2043.0 | 2376.8 |
|  |  |  |  | (2.3) | (159.5) | (227.2) | (289.5) | (289.2) | (189.7) |
|  |  |  |  |  |  |  |  |  |  |
| Chaperone (Structural) | YscG homolog | *vp1693* | Class III chaperone | 18.9 | 1238.1 | 1053.5 | 1196.0 | 1246.0 | 1218.5 |
|  |  |  |  | (1.6) | (56.5) | (80.1) | (131.1) | (236.2) | (59.5) |
|  | YscW homolog | *vp1700* | Pilotin lipoprotein | 27.6 | 138.6 | 333.6 | 414.7 | 532.3 | 503.9 |
|  |  |  |  | (1.6) | (34.8) | (58.5) | (31.3) | (55.5) | (83.9) |
|  |  |  |  |  |  |  |  |  |  |
| Not determined | YscY homolog | *vp1663* | Putative YscX chaperone | 2.4 | 245.5 | 476.3 | 793.6 | 876.2 | 1010.5 |
|  |  |  |  | (0.1) | (24.6) | (42.1) | (136.2) | (29.8) | (45.8) |
|  | YscX homolog | *vp1664* | Unknown | 7.6 | 241.4 | 599.1 | 1068.2 | 1453.7 | 1504.2 |
|  |  |  |  | (0.9) | (31.0) | (22.4) | (45.6) | (117.8) | (110.9) |
|  | SycN homolog | *vp1665* | YopN/SycN/YscB/TyeA complex | 16.3 | 244.2 | 602.0 | 1027.2 | 1547.0 | 1841.6 |
|  |  |  |  | (3.0) | (19.0) | (48.6) | (10.8) | (118.3) | (120.2) |
|  | TyeA homolog | *vp1666* | YopN/SycN/YscB/TyeA complex | 30.4 | 375.8 | 1066.0 | 2114.1 | 2923.7 | 2953.8 |
|  |  |  |  | (3.6) | (11.8) | (219.6) | (10.7) | (185.8) | (295.9) |
|  | YopN homolog | *vp1667* | YopN/SycN/YscB/TyeA complex | 49.2 | 443.4 | 1194.8 | 2262.6 | 2984.2 | 3254.9 |
|  |  |  |  | (0.0) | (26.3) | (117.2) | (93.2) | (125.3) | (312.6) |
|  | YscO homolog | *vp1669* | Unknown | 8.9 | 134.7 | 415.3 | 643.1 | 895.5 | 994.5 |
|  |  |  |  | (0.6) | (34.6) | (31.9) | (110.2) | (85.3) | (139.2) |
|  |  | *vp1676* | Putative LysR-family transcriptional regulator | 10.0 | 60.8 | 55.8 | 63.6 | 50.2 | 55.6 |
|  |  |  |  | (0.9) | (5.6) | (8.8) | (4.0) | (12.8) | (1.2) |
|  | Hypothetical protein | *vp1677* | Unknown | 20.2 | 21.1 | 38.5 | 48.1 | 72.8 | 46.7 |
|  |  |  |  | (1.2) | (3.2) | (7.9) | (3.6) | (27.2) | (1.7) |
|  |  | *vp1678* | Putative dienelactone hydrolase and related enzymes | 22.5 | 34.7 | 45.0 | 73.2 | 76.7 | 65.6 |
|  |  |  |  | (2.8) | (10.7) | (11.5) | (4.3) | (14.5) | (14.4) |
|  | Hypothetical protein | *vp1679* | Unknown | 7.0 | 28.7 | 44.1 | 54.3 | 71.3 | 61.0 |
|  |  |  |  | (1.0) | (5.1) | (8.7) | (3.8) | (13.4) | (7.9) |
|  | Hypothetical protein | *vp1681* | Unknown | 0.0 | 7.6 | 5.2 | 5.8 | 12.0 | 3.5 |
|  |  |  |  | (0.0) | (4.0) | (0.5) | (5.8) | (9.4) | (1.4) |
|  | CesT family chaperone | *vp1684* | Unknown | 22.4 | 133.3 | 270.9 | 413.9 | 596.3 | 617.7 |
|  |  |  |  | (1.7) | (12.2) | (30.7) | (12.2) | (108.9) | (5.6) |
|  | Hypothetical protein | *vp1685* | Unknown | 2.1 | 18.3 | 47.3 | 83.0 | 115.6 | 137.3 |
|  |  |  |  | (2.1) | (4.9) | (1.8) | (3.8) | (34.0) | (3.4) |
|  | YscL homolog | *vp1688* | Interactor of ATPase/C ring | 6.5 | 510.6 | 335.8 | 463.8 | 440.0 | 432.0 |
|  |  |  |  | (1.4) | (39.0) | (89.3) | (25.3) | (159.2) | (58.5) |
|  | YscK homolog | *vp1689* | Interactor of ATPase/C ring | 5.0 | 567.0 | 450.6 | 525.8 | 547.1 | 567.8 |
|  |  |  |  | (0.7) | (11.6) | (106.7) | (20.7) | (178.7) | (92.5) |
|  | YscH homolog | *vp1692* | Encodes YopR - Unknown function | 12.9 | 847.8 | 848.7 | 1010.4 | 1059.6 | 1121.6 |
|  |  |  |  | (2.9) | (201.9) | (45.0) | (221.5) | (172.3) | (28.3) |
|  | YscB homolog | *vp1697* | YopN/SycN/YscB/TyeA complex | 19.7 | 684.2 | 1416.3 | 2594.7 | 2917.3 | 3109.5 |
|  |  |  |  | (0.6) | (151.3) | (290.9) | (289.9) | (259.4) | (0.0) |
|  |  |  |  |  |  |  |  |  |  |
