# Supplementary material for: Transcriptome analysis of Vibrio parahaemolyticus in type III secretion system 1 inducing conditions
Source: Front Cell Infect Microbiol. 2014 Jan 20;4:1. doi: 10.3389/fcimb.2014.00001 (PMC3895804; doi:10.3389/fcimb.2014.00001)
Supplement: Supplementary file 3 [file DataSheet3.DOCX]

Supplementary Table 3. Genes showing ≥5-fold increase in expression (*P*< 0.05) in the DMEM condition when compared to the LB-S condition (see Material and Methods).

| **Locus Tag** | **Gene** | **Putative Product** | **COG** | **Fold Change** | ***P*-value** |
| --- | --- | --- | --- | --- | --- |
| *vp0008* | - | amino acid ABC transporter substrate-binding protein | COG0834ET | 25.8 | 0.00 |
| *vp0074* | - | hypothetical protein | COG0056C | 51.7 | 0.00 |
| *vp0106* | - | hypothetical protein | - | 325.4 | 0.00 |
| *vp0182* | - | hypothetical protein | - | 5.4 | 0.02 |
| *vp0239* | tpiA | triosephosphate isomerase | COG0149G | 8.6 | 0.00 |
| *vp0345* | - | hypothetical protein | - | 5.7 | 0.00 |
| *vp0356* | - | pyruvate kinase | COG0469G | 8.2 | 0.00 |
| *vp0357* | - | hypothetical protein | - | 6.1 | 0.00 |
| *vp0483* | gltD | glutamate synthase subunit beta | COG0493ER | 26.6 | 0.00 |
| *vp0484* | - | glutamate synthase, large subunit | COG0069E | 61.5 | 0.00 |
| *vp0485* | - | hypothetical protein | COG1242R | 8.0 | 0.00 |
| *vp0494* | thrA | bifunctional aspartokinase I/homoserine dehydrogenase I | COG0527E | 9.6 | 0.00 |
| *vp0495* | - | homoserine kinase | COG0083E | 10.4 | 0.00 |
| *vp0496* | - | threonine synthase | COG0498E | 18.6 | 0.00 |
| *vp0497* | - | autonomous glycyl radical cofactor GrcA | COG3445R | 18.5 | 0.00 |
| *vp0516* | - | aldo/keto reductase | COG0667C | 6.4 | 0.00 |
| *vp0525* | - | hypothetical protein | COG3930S | 7.9 | 0.00 |
| *vp0540* | - | carbon starvation protein A | COG1966T | 111.2 | 0.02 |
| *vp0552* | - | soluble lytic murein transglycosylase | COG0741M | 5.6 | 0.00 |
| *vp0582* | - | hypothetical protein | - | 35.6 | 0.00 |
| *vp0583* | - | malate synthase | COG2225C | 8.5 | 0.00 |
| *vp0669* | - | hypothetical protein | COG3608R | 14.8 | 0.00 |
| *vp0704* | metQ | DL-methionine transporter substrate-binding subunit | COG1464P | 8.5 | 0.00 |
| *vp0705* | - | ABC transporter permease | COG2011P | 7.5 | 0.00 |
| *vp0706* | metN | DL-methionine transporter ATP-binding subunit | COG1135P | 9.4 | 0.00 |
| *vp0765* | - | hypothetical protein | - | 13.8 | 0.00 |
| *vp0766* | - | hypothetical protein | - | 11.4 | 0.00 |
| *vp0794* | - | phosphoenolpyruvate-protein phosphotransferase | COG1080G | 6.2 | 0.00 |
| *vp0810* | - | PTS system mannose-specific, factor IIC | - | 14.8 | 0.00 |
| *vp0830* | - | hypothetical protein | - | 6.0 | 0.00 |
| *vp0857* | - | ferrous iron transport protein A | COG1918P | 46.1 | 0.00 |
| *vp0858* | - | ferrous iron transport protein B | COG0370P | 39.9 | 0.00 |
| *vp0859* | - | hypothetical protein | - | 26.0 | 0.00 |
| *vp0864* | purU | formyltetrahydrofolate deformylase | COG0788F | 5.1 | 0.00 |
| *vp0971* | - | NADH dehydrogenase | COG1252C | 6.2 | 0.00 |
| *vp0994* | - | formate acetyltransferase | COG1882C | 5.7 | 0.00 |
| *vp0995* | - | hypothetical protein | - | 10.6 | 0.00 |
| *vp1008* | - | outer membrane porin protein | COG3203M | 25.7 | 0.00 |
| *vp1012* | - | cold shock-like protein CspD | COG1278K | 6.4 | 0.00 |
| *vp1070* | - | hypothetical protein | - | 8.3 | 0.00 |
| *vp1112* | - | adenosylmethionine-8-amino-7-oxononanoate aminotransferase | COG0161H | 45.0 | 0.00 |
| *vp1113* | - | biotin synthase | COG0502H | 23.2 | 0.00 |
| *vp1114* | - | 8-amino-7-oxononanoate synthase | COG0156H | 12.4 | 0.00 |
| *vp1115* | - | biotin synthesis protein BioC | COG2226H | 7.0 | 0.00 |
| *vp1122* | - | hypothetical protein | COG3496S | 5.6 | 0.00 |
| *vp1125* | - | hypothetical protein | - | 7.8 | 0.00 |
| *vp1164* | - | hypothetical protein | - | 436.0 | 0.00 |
| *vp1221* | - | cytochrome subunit of sulfide dehydrogenase | COG2863C | 6.9 | 0.00 |
| *vp1332* | - | binding protein component of ABC transporter | COG0687E | 7.0 | 0.01 |
| *vp1361* | - | ABC transport system permease | COG0767Q | 5.7 | 0.00 |
| *vp1363* | - | ABC transporter ATP-binding protein | COG1127Q | 5.1 | 0.00 |
| *vp1364* | - | ABC transporter periplasmic substrate-binding protein | COG1463Q | 8.4 | 0.00 |
| *vp1437* | - | hypothetical protein | - | 5.6 | 0.00 |
| *vp1480* | - | riboflavin synthase subunit alpha | COG0307H | 8.3 | 0.00 |
| *vp1566* | - | structural protein P5 | - | 5.0 | 0.02 |
| *vp1620* | - | amino acid ABC transporter substrate-binding protein | COG0834ET | 8.3 | 0.00 |
| *vp1621* | - | amino acid ABC transporter permease | COG4597E | 5.7 | 0.00 |
| *vp1622* | - | amino acid ABC transporter permease | COG0765E | 6.5 | 0.00 |
| *vp1640* | - | hypothetical protein | COG0561R | 6.4 | 0.00 |
| *vp1656* | YopD homolog | hydrophobic translocator | - | 12.1 | 0.00 |
| *vp1657* | YopB homolog | hydrophobic translocator | COG5613S | 14.4 | 0.00 |
| *vp1658* | LcrH homolog | class II translocator chaperone | COG5010U | 15.4 | 0.00 |
| *vp1659* | LcrV homolog | hydrophilic translocator, injectisome tip | - | 12.5 | 0.00 |
| *vp1660* | LcrG homolog | LcrV chaperone, negative regulator of effector secretion | - | 24.1 | 0.00 |
| *vp1661* | LcrR homolog | regulator, low calcium response protein | - | 12.1 | 0.00 |
| *vp1662* | YscV homolog | inner Membrane export apparatus | COG4789U | 18.7 | 0.00 |
| *vp1663* | YscY homolog | putative YscX chaperone | COG4783R | 23.3 | 0.00 |
| *vp1664* | YscX homolog | unknown | - | 45.2 | 0.00 |
| *vp1665* | SycN homolog | YopN/SycN/YscB/TyeA complex | - | 25.4 | 0.00 |
| *vp1666* | TyeA homolog | YopN/SycN/YscB/TyeA complex | - | 34.1 | 0.00 |
| *vp1667* | YopN homolog | YopN/SycN/YscB/TyeA complex | - | 35.0 | 0.00 |
| *vp1668* | YscN homolog | ATPase | COG1157NU | 27.4 | 0.00 |
| *vp1669* | YscO homolog | unknown | - | 25.4 | 0.00 |
| *vp1670* | YscP homolog | ruler - needle length control, substrate specificity switch | - | 26.4 | 0.00 |
| *vp1671* | YscQ homolog | cytoplasmic ring - sorting platform for T3S cargo proteins | COG1886NU | 43.4 | 0.00 |
| *vp1672* | YscR homolog | inner membrane export apparatus | COG4790U | 27.5 | 0.00 |
| *vp1673* | YscS homolog | inner membrane export apparatus | COG4794U | 12.5 | 0.00 |
| *vp1674* | YscT homolog | inner membrane export apparatus | COG4791U | 7.8 | 0.00 |
| *vp1675* | YscU homolog | inner membrane export apparatus | COG4792U | 5.1 | 0.00 |
| *vp1680* | VopQ/VepA | autophagy effector protein | - | 6.0 | 0.00 |
| *vp1682* | VopQ chaperone/VecA | VopQ chaperone | - | 21.1 | 0.00 |
| *vp1683* | VopR | unknown - putative effector protein | - | 13.2 | 0.00 |
| *vp1686* | VopS | Rho GTPase inhibition effector protein, actin rearrangement | COG3177S | 35.6 | 0.00 |
| *vp1687* | VopS chaperone | putative VopS chaperone | - | 49.3 | 0.00 |
| *vp1688* | YscL homolog | interactor of ATPase/C ring | COG1317NU | 8.8 | 0.00 |
| *vp1690* | YscJ homolog | membrane and supramembrane (MS) ring | COG4669U | 8.6 | 0.00 |
| *vp1691* | YscI homolog | inner rod protein | - | 7.0 | 0.00 |
| *vp1692* | YscH homolog | encodes YopR - unknown function | - | 11.9 | 0.00 |
| *vp1693* | YscG homolog | class III chaperone | - | 10.7 | 0.00 |
| *vp1694* | YscF homolog | needle protein | - | 12.4 | 0.00 |
| *vp1695* | YscD homolog | membrane and supramembrane (MS) ring | - | 9.3 | 0.00 |
| *vp1696* | YscC homolog | outer membrane secretin ring | COG1450NU | 10.4 | 0.00 |
| *vp1697* | YscB homolog | YopN/SycN/YscB/TyeA complex | - | 18.6 | 0.00 |
| *vp1698* | ExsD | negative regulator of T3SS1 activity | - | 31.2 | 0.00 |
| *vp1700* | YscW homolog | pilotin lipoprotein | - | 58.5 | 0.00 |
| *vp1701* | ExsC | putative ExsD inhibitor | - | 15.0 | 0.00 |
| *vp1702* | ExsE | putative ExsC inhibitor | - | 15.6 | 0.00 |
| *vp1723* | - | BCCT family transporter | COG1292M | 7.7 | 0.00 |
| *vp1743* | - | hypothetical protein | COG1280E | 6.3 | 0.00 |
| *vp1747* | - | amino acid transporter | COG0531E | 6.9 | 0.00 |
| *vp1779* | - | glutamine amidotransferase | COG2071R | 11.6 | 0.00 |
| *vp1781* | - | glutamine synthetase | COG0174E | 16.7 | 0.00 |
| *vp1897* | - | hypothetical protein | - | 68.1 | 0.00 |
| *vp1898* | - | hypothetical protein | - | 5.6 | 0.00 |
| *vp1899* | - | hypothetical protein | - | 6.3 | 0.02 |
| *vp1900* | - | aromatic amino acid aminotransferase | COG1448E | 23.0 | 0.00 |
| *vp1916* | - | amidase | COG2989S | 6.2 | 0.00 |
| *vp1917* | - | hypothetical protein | COG3490S | 21.4 | 0.00 |
| *vp1918* | - | hypothetical protein | COG3489R | 24.1 | 0.00 |
| *vp1919* | - | hypothetical protein | COG3488C | 40.7 | 0.00 |
| *vp1920* | - | iron-regulated protein A | COG3487P | 135.9 | 0.00 |
| *vp1999* | - | hypothetical protein | COG0845M | 7.5 | 0.00 |
| *vp2003* | - | hypothetical protein | COG4976 | 5.8 | 0.05 |
| *vp2004* | - | hypothetical protein | - | 6.7 | 0.00 |
| *vp2110* | - | hypothetical protein | - | 107.0 | 0.00 |
| *vp2121* | - | bifunctional acetaldehyde-CoA/alcohol dehydrogenase | COG1454C | 8.4 | 0.02 |
| *vp2124* | - | aspartate-semialdehyde dehydrogenase | COG0136E | 7.5 | 0.00 |
| *vp2145* | - | histone deacetylase/AcuC/AphA family protein | COG0123BQ | 12.4 | 0.00 |
| *vp2157* | - | glyceraldehyde-3-phosphate dehydrogenase | COG0057G | 24.5 | 0.00 |
| *vp2158* | - | hypothetical protein | COG0676G | 6.4 | 0.00 |
| *vp2161* | - | hypothetical protein | COG3016S | 22.3 | 0.00 |
| *vp2166* | - | lactoylglutathione lyase | COG0346E | 32.9 | 0.00 |
| *vp2167* | - | hypothetical protein | COG1359S | 20.5 | 0.00 |
| *vp2331* | rpmE2 | 50S ribosomal protein L31 | COG0254J | 66.5 | 0.00 |
| *vp2335* | - | hypothetical protein | - | 8.4 | 0.00 |
| *vp2336* | - | hypothetical protein | - | 7.1 | 0.00 |
| *vp2371* | - | N-acetylglutamate synthase | COG0548E | 8.8 | 0.00 |
| *vp2420* | - | pilus assembly protein | COG3745U | 6.4 | 0.00 |
| *vp2422* | - | hypothetical protein | COG4960OU | 6.0 | 0.00 |
| *vp2423* | - | fimbrial protein | COG3847U | 15.8 | 0.00 |
| *vp2431* | serB | phosphoserine phosphatase | COG0560E | 5.0 | 0.00 |
| *vp2489* | - | iron(III) ABC transporter ATP-binding protein | COG3842E | 8.4 | 0.00 |
| *vp2490* | - | iron(III) ABC transporter permease | COG1178P | 7.2 | 0.00 |
| *vp2491* | - | iron(III) ABC transporter periplasmic iron-compound-binding protein | COG1840P | 31.0 | 0.00 |
| *vp2593* | - | D-3-phosphoglycerate dehydrogenase | COG0111HE | 29.3 | 0.00 |
| *vp2596* | - | LysE/YggA family protein | COG1279R | 7.9 | 0.00 |
| *vp2597* | - | DNA-binding protein | COG2606S | 22.7 | 0.00 |
| *vp2600* | pgk | phosphoglycerate kinase | COG0126G | 5.3 | 0.00 |
| *vp2602* | - | enterobactin receptor protein | COG4771P | 17.4 | 0.00 |
| *vp2603* | - | iron-regulated virulence regulatory protein | COG0583K | 41.9 | 0.00 |
| *vp2647* | - | hypothetical protein | COG3205S | 15.1 | 0.00 |
| *vp2653* | - | ornithine carbamoyltransferase | COG0078E | 24.5 | 0.00 |
| *vp2723* | - | hypothetical protein | - | 9.7 | 0.00 |
| *vp2731* | pgi | glucose-6-phosphate isomerase | COG0166G | 11.5 | 0.00 |
| *vp2756* | - | bifunctional argininosuccinate lyase/N-acetylglutamate synthase | COG0165E | 6.0 | 0.00 |
| *vp2757* | - | argininosuccinate synthase | COG0137E | 13.0 | 0.00 |
| *vp2758* | - | acetylglutamate kinase | COG0548E | 65.0 | 0.00 |
| *vp2759* | argC | N-acetyl-gamma-glutamyl-phosphate reductase | COG0002E | 113.7 | 0.00 |
| *vp2761* | - | phosphoenolpyruvate carboxylase | COG2352C | 17.2 | 0.00 |
| *vp2763* | metF | 5,10-methylenetetrahydrofolate reductase | COG0685E | 5.4 | 0.00 |
| *vp2768* | - | bacterioferritin | COG2193P | 39.5 | 0.00 |
| *vp2769* | - | bacterioferritin-associated ferredoxin | COG2906P | 88.2 | 0.00 |
| *vp2829* | - | phosphoglyceromutase | COG0696G | 20.7 | 0.00 |
| *vp2860* | - | superoxide dismutase, Mn | COG0605P | 77.9 | 0.00 |
| *vp2861* | - | rRNA methylase | COG0219J | 19.6 | 0.00 |
| *vp2872* | - | hypothetical protein | - | 154.3 | 0.00 |
| *vp2873* | fumC | fumarate hydratase | COG0114C | 216.6 | 0.00 |
| *vp2944* | - | O-methyltransferase-like protein | COG3315Q | 25.5 | 0.00 |
| *vp2963* | - | hypothetical protein | - | 40.5 | 0.01 |
| *vpa0003* | - | peptide ABC transporter ATP-binding protein | COG1123R | 9.9 | 0.00 |
| *vpa0004* | - | peptide ABC transporter ATP-binding protein | COG0444EP | 10.4 | 0.00 |
| *vpa0035* | - | sodium/glutamate symporter | COG0786E | 10.8 | 0.00 |
| *vpa0037* | - | hypothetical protein | - | 6.7 | 0.01 |
| *vpa0040* | - | hypothetical protein | COG3085S | 303.9 | 0.00 |
| *vpa0044* | - | hypothetical protein | - | 380.9 | 0.00 |
| *vpa0045* | - | ada regulatory protein | COG2169F | 6.7 | 0.00 |
| *vpa0046* | - | methylated-DNA-protein-cysteine S-methyltransferase | COG0350L | 5.9 | 0.00 |
| *vpa0048* | - | hypothetical protein | - | 5.6 | 0.02 |
| *vpa0057* | - | hypothetical protein | COG3123S | 5.2 | 0.00 |
| *vpa0089* | - | siderophore utilization protein | COG2375P | 19.3 | 0.00 |
| *vpa0103* | - | hypothetical protein | COG2110R | 12.4 | 0.00 |
| *vpa0137* | - | hypothetical protein | - | 252.9 | 0.00 |
| *vpa0144* | - | D-lactate dehydrogenase | COG1052CHR | 17.9 | 0.00 |
| *vpa0147* | - | lactate dehydrogenase | COG0039C | 21.1 | 0.00 |
| *vpa0150* | - | ferrichrome-iron receptor | COG4773P | 47.1 | 0.00 |
| *vpa0151* | - | hypothetical protein | - | 28.5 | 0.00 |
| *vpa0152* | - | biopolymer transport protein ExbB-like protein | COG0811U | 22.7 | 0.00 |
| *vpa0153* | - | TonB system transport protein ExbB2 | COG0811U | 16.9 | 0.00 |
| *vpa0154* | - | TonB system transport protein ExbD2 | COG0848U | 21.0 | 0.00 |
| *vpa0155* | - | TonB2 protein | COG0810M | 20.7 | 0.00 |
| *vpa0156* | - | hypothetical protein | COG2956G | 22.7 | 0.00 |
| *vpa0157* | - | hypothetical protein | COG3295S | 6.4 | 0.00 |
| *vpa0158* | - | hypothetical protein | COG3656S | 31.3 | 0.00 |
| *vpa0159* | - | hypothetical protein | COG5266P | 8.0 | 0.00 |
| *vpa0213* | - | hypothetical protein | - | 8.4 | 0.00 |
| *vpa0255* | - | hypothetical protein | COG1434S | 6.0 | 0.00 |
| *vpa0286* | groES | co-chaperonin GroES | COG0234O | 66.5 | 0.00 |
| *vpa0287* | groEL | molecular chaperone GroEL | COG0459O | 53.7 | 0.00 |
| *vpa0289* | - | hypothetical protein | COG1280E | 19.8 | 0.00 |
| *vpa0302* | - | hypothetical protein | COG0346E | 5.3 | 0.00 |
| *vpa0310* | - | hypothetical protein | - | 67.6 | 0.00 |
| *vpa0311* | - | hypothetical protein | - | 33.7 | 0.03 |
| *vpa0321* | - | hypothetical protein | COG1342R | 6.6 | 0.00 |
| *vpa0323* | - | hypothetical protein | COG1433S | 5.9 | 0.00 |
| *vpa0324* | - | hypothetical protein | COG2991S | 6.6 | 0.00 |
| *vpa0358* | - | LuxR family transcriptional regulator | COG2197TK | 8.1 | 0.00 |
| *vpa0359* | - | hypothetical protein | COG1476K | 5.9 | 0.00 |
| *vpa0367* | - | spermidine/putrescine ABC transporter periplasmic spermidine/putrescine-binding protein | COG0687E | 21.3 | 0.00 |
| *vpa0391* | - | hypothetical protein | - | 5.1 | 0.00 |
| *vpa0421* | hmuV | hemin importer ATP-binding subunit | COG4559P | 108.1 | 0.00 |
| *vpa0422* | - | hemin ABC transporter permease | COG0609P | 257.1 | 0.00 |
| *vpa0423* | - | hemin ABC transporter periplasmic hemin-binding protein HutB | COG4558P | 598.8 | 0.00 |
| *vpa0424* | - | TonB system transport protein ExbD1 | COG0848U | 729.6 | 0.00 |
| *vpa0425* | - | ExbB-like protein | COG0811U | 1155.9 | 0.00 |
| *vpa0426* | - | TonB-like protein | COG0810M | 223.8 | 0.00 |
| *vpa0427* | - | coproporphyrinogen III oxidase | COG0635H | 536.4 | 0.00 |
| *vpa0428* | - | hypothetical protein | COG3721P | 140.6 | 0.00 |
| *vpa0429* | - | hypothetical protein | COG0748P | 208.9 | 0.00 |
| *vpa0430* | - | hypothetical protein | COG2096S | 5.2 | 0.00 |
| *vpa0448* | - | hypothetical protein | - | 6.1 | 0.00 |
| *vpa0450* | VPA0450 | inositol phosphatase effector protein | - | 11.7 | 0.00 |
| *vpa0454* | - | DNA-binding stress protein | COG0783P | 9.6 | 0.00 |
| *vpa0474* | - | spermidine n1-acetyltransferase | COG1670J | 5.3 | 0.00 |
| *vpa0475* | - | hypothetical protein | - | 78.0 | 0.00 |
| *vpa0523* | - | hypothetical protein | - | 23.3 | 0.00 |
| *vpa0535* | - | phosphomannomutase | COG1109G | 8.8 | 0.00 |
| *vpa0592* | - | hypothetical protein | - | 74.6 | 0.00 |
| *vpa0593* | - | transcriptional regulator | COG1414K | 55.2 | 0.00 |
| *vpa0597* | - | hypothetical protein | - | 8.3 | 0.01 |
| *vpa0627* | - | cytochrome o ubiquinol oxidase subunit II | COG1622C | 15.3 | 0.00 |
| *vpa0635* | - | oxidoreductase, oxygen dependent, FAD-dependent protein | COG0277C | 10.7 | 0.03 |
| *vpa0636* | artP | arginine transporter ATP-binding subunit | COG4161E | 343.1 | 0.00 |
| *vpa0637* | - | arginine ABC transporter substrate-binding protein | COG0834ET | 47.0 | 0.00 |
| *vpa0638* | - | arginine transporter permease subunit ArtQ | COG4215E | 30.6 | 0.00 |
| *vpa0639* | artM | arginine transporter permease subunit ArtM | COG4160E | 10.3 | 0.00 |
| *vpa0641* | - | LysR family transcriptional regulator | COG0583K | 5.2 | 0.00 |
| *vpa0642* | - | glutathione S-transferase | COG0625O | 11.3 | 0.00 |
| *vpa0657* | - | iron(III) ABC transporter periplasmic iron-compound-binding protein | COG4607P | 106.9 | 0.00 |
| *vpa0658* | - | iron(III) ABC transporter permease | COG4606P | 96.7 | 0.00 |
| *vpa0659* | - | iron(III) ABC transporter permease | COG4605P | 49.2 | 0.00 |
| *vpa0660* | - | iron(III) ABC transporter ATP-binding protein | COG4604P | 39.2 | 0.00 |
| *vpa0661* | - | hypothetical protein | - | 6.8 | 0.00 |
| *vpa0663* | - | AraC family transcriptional regulator | COG2207K | 59.8 | 0.00 |
| *vpa0664* | - | Fe-regulated protein B | COG4771P | 673.8 | 0.00 |
| *vpa0665* | - | hypothetical protein | - | 96.1 | 0.00 |
| *vpa0667* | - | hypothetical protein | - | 122.4 | 0.00 |
| *vpa0678* | - | hypothetical protein | COG0745TK | 21.2 | 0.00 |
| *vpa0680* | - | arylsulfatase | COG3119P | 8.1 | 0.00 |
| *vpa0681* | - | hypothetical protein | COG4783R | 5.9 | 0.00 |
| *vpa0694* | - | hypothetical protein | COG3111S | 11.5 | 0.00 |
| *vpa0695* | - | accessory colonization factor AcfA | - | 9.6 | 0.00 |
| *vpa0745* | - | hypothetical protein | - | 26.4 | 0.03 |
| *vpa0796* | - | L-allo-threonine aldolase | COG2008E | 7.4 | 0.00 |
| *vpa0851* | - | formate transporter 1 | COG2116P | 493.6 | 0.00 |
| *vpa0882* | - | heme transport protein HutA | COG1629P | 365.8 | 0.00 |
| *vpa0894* | - | hypothetical protein | - | 11.8 | 0.01 |
| *vpa0922* | pntA | NAD(P) transhydrogenase subunit alpha | COG3288C | 5.6 | 0.00 |
| *vpa0933* | - | hypothetical protein | COG3111S | 17.6 | 0.01 |
| *vpa0959* | - | formate dehydrogenase oxidoreductase protein | COG0243C | 33.8 | 0.00 |
| *vpa0960* | - | hypothetical protein | - | 195.7 | 0.02 |
| *vpa0961* | - | transcriptional regulator | COG0583K | 224.0 | 0.00 |
| *vpa0962* | - | formate dehydrogenase oxidoreductase protein | COG1526C | 117.1 | 0.00 |
| *vpa0973* | - | MFS family transporter | COG2814G | 33.9 | 0.00 |
| *vpa0979* | - | ferric aerobactin receptor | COG1629P | 112.0 | 0.00 |
| *vpa0980* | - | hypothetical protein | COG4114R | 34.9 | 0.00 |
| *vpa1011* | - | L-allo-threonine aldolase | COG2008E | 5.1 | 0.00 |
| *vpa1048* | - | anti-sigma F factor antagonist | COG2172T | 6.1 | 0.00 |
| *vpa1049* | - | two-component response regulator | COG2208TK | 6.4 | 0.00 |
| *vpa1050* | - | hypothetical protein | - | 13.0 | 0.00 |
| *vpa1078* | - | hypothetical protein | - | 5.8 | 0.00 |
| *vpa1087* | - | D-ribose pyranase | COG1869G | 7.3 | 0.00 |
| *vpa1091* | - | hypothetical protein | COG5178A | 5.5 | 0.00 |
| *vpa1108* | - | NAD(P)H-flavin reductase | COG0778C | 17.8 | 0.00 |
| *vpa1130* | - | sensory box sensor histidine kinase/response regulator | COG0642T | 8.5 | 0.00 |
| *vpa1131* | - | periplasmic binding protein-like protein | COG3221P | 10.7 | 0.00 |
| *vpa1143* | - | molybdenum containing oxidoreductase | COG2041R | 5.5 | 0.00 |
| *vpa1144* | - | hypothetical protein | COG3474C | 7.3 | 0.00 |
| *vpa1163* | - | aldo/keto reductase | COG4989R | 5.4 | 0.00 |
| *vpa1166* | - | chloride channel protein | COG0038P | 12.7 | 0.00 |
| *vpa1183* | - | hypothetical protein | - | 6.6 | 0.02 |
| *vpa1226* | - | hypothetical protein | COG2926S | 8.9 | 0.00 |
| *vpa1273* | - | hypothetical protein | - | 9.0 | 0.00 |
| *vpa1275* | - | short chain dehydrogenase/reductase family oxidoreductase | COG4221R | 10.6 | 0.00 |
| *vpa1285* | - | hypothetical protein | - | 65.1 | 0.00 |
| *vpa1287* | - | transporter | COG1230P | 20.7 | 0.00 |
| *vpa1305* | - | hypothetical protein | - | 17.8 | 0.02 |
| *vpa1387* | - | hypothetical protein | - | 6.0 | 0.00 |
| *vpa1388* | - | hypothetical protein | - | 5.5 | 0.00 |
| *vpa1395* | - | transposase | - | 9.1 | 0.00 |
| *vpa1396* | - | hypothetical protein | - | 15.9 | 0.00 |
| *vpa1400* | malF | maltose transporter membrane protein | COG1175G | 7.1 | 0.00 |
| *vpa1402* | - | maltose/maltodextrin transporter ATP-binding protein | COG3839G | 8.4 | 0.00 |
| *vpa1418* | - | catalase | COG0753P | 10.5 | 0.00 |
| *vpa1423* | - | transcriptional regulator | COG2207K | 7.7 | 0.00 |
| *vpa1429* | - | hypothetical protein | COG2200T | 9.0 | 0.00 |
| *vpa1434* | - | hemolysin secretion ATP-binding protein | COG1132V | 6.3 | 0.00 |
| *vpa1435* | - | iron(III) compound receptor | COG4773P | 75.2 | 0.00 |
| *vpa1436* | - | iron(III) ABC transporter ATP-binding protein | COG1120PH | 64.8 | 0.00 |
| *vpa1437* | - | iron(III) ABC transporter periplasmic iron-compound-binding protein | COG0614P | 11.3 | 0.00 |
| *vpa1438* | - | iron-hydroxamate transporter permease subunit | COG0609P | 16.9 | 0.00 |
| *vpa1446* | - | LuxR family transcriptional regulator | COG2197TK | 9.0 | 0.01 |
| *vpa1463* | - | hypothetical protein | - | 62.1 | 0.00 |
| *vpa1464* | - | hypothetical protein | - | 57.3 | 0.00 |
| *vpa1465* | - | hypothetical protein | COG4935O | 75.7 | 0.00 |
| *vpa1466* | - | TonB system receptor | COG4771P | 115.2 | 0.00 |
| *vpa1467* | - | protease II | COG1770E | 128.0 | 0.00 |
| *vpa1494* | - | hypothetical protein | - | 280.3 | 0.00 |
| *vpa1495* | - | ABC transporter ATP-binding protein | COG4172R | 589.0 | 0.00 |
| *vpa1502* | - | carbonic anhydrase | COG3338P | 25.4 | 0.00 |
| *vpa1507* | - | CsuA protein | COG5430S | 5.3 | 0.00 |
| *vpa1514* | - | superoxide dismutase, Cu-Zn | COG2032P | 9.3 | 0.00 |
| *vpa1528* | - | hypothetical protein | COG3832S | 44.2 | 0.00 |
| *vpa1529* | - | hypothetical protein | COG5006R | 60.7 | 0.00 |
| *vpa1531* | - | serine protease | COG5640O | 8.1 | 0.00 |
| *vpa1560* | - | hypothetical protein | - | 18.6 | 0.00 |
| *vpa1577* | - | hypothetical protein | - | 7.5 | 0.00 |
| *vpa1590* | - | hypothetical protein | COG1359S | 6.3 | 0.00 |
| *vpa1591* | - | dihydropteridine reductase | COG0778C | 6.3 | 0.00 |
| *vpa1594* | - | D-alanyl-D-alanine carboxypeptidase | COG1686M | 5.8 | 0.00 |
| *vpa1599* | - | hypothetical protein | COG4667R | 8.5 | 0.00 |
| *vpa1619* | malQ | 4-alpha-glucanotransferase | COG1640G | 8.1 | 0.00 |
| *vpa1620* | - | maltodextrin phosphorylase | COG0058G | 35.0 | 0.00 |
| *vpa1650* | - | insulinase family zinc protease | COG0612R | 8.0 | 0.00 |
| *vpa1652* | fecE | iron-dicitrate transporter ATP-binding subunit | COG1120PH | 49.9 | 0.00 |
| *vpa1653* | - | ferrichrome ABC transporter permease | COG0609P | 119.9 | 0.00 |
| *vpa1654* | - | ferrichrome ABC transporter permease | COG0609P | 58.5 | 0.00 |
| *vpa1655* | fecB | iron-dicitrate transporter substrate-binding subunit | COG4594P | 79.9 | 0.00 |
| *vpa1656* | - | ferric vibrioferrin receptor | COG4772P | 654.0 | 0.00 |
| *vpa1657* | - | ferric siderophore receptor-like protein | COG4774P | 345.6 | 0.00 |
| *vpa1658* | - | hypothetical protein | COG0439I | 581.7 | 0.00 |
| *vpa1659* | - | hypothetical protein | COG4264Q | 514.7 | 0.00 |
| *vpa1660* | - | transport protein | COG2814G | 443.6 | 0.00 |
| *vpa1661* | - | AcsD | COG4264Q | 419.1 | 0.00 |
| *vpa1662* | - | diaminopimelate decarboxylase protein | COG0019E | 167.0 | 0.00 |
| *vpa1668* | - | hypothetical protein | - | 26.9 | 0.00 |
| *vpa1700* | - | mannonate dehydratase | COG1312G | 74.2 | 0.00 |
| *vpa1703* | - | small integral C4-dicarboxylate membrane transport protein | COG3090G | 18.3 | 0.00 |
| *vpa1704* | - | integral membrane protein transporter | COG1593G | 20.4 | 0.00 |
| *vpa1705* | - | mannonate oxidoreductase | COG0246G | 22.5 | 0.00 |
| *vpa1706* | - | glucuronate isomerase | COG1904G | 19.8 | 0.00 |
| *vpa1707* | - | 2-dehydro-3-deoxygluconokinase | COG0524G | 19.3 | 0.00 |
| *vpa1708* | - | keto-hydroxyglutarate-aldolase/keto-deoxy-phosphogluconate aldolase | COG0800G | 16.0 | 0.00 |
