# Supplementary material for: Transcriptome analysis of Vibrio parahaemolyticus in type III secretion system 1 inducing conditions
Source: Front Cell Infect Microbiol. 2014 Jan 20;4:1. doi: 10.3389/fcimb.2014.00001 (PMC3895804; doi:10.3389/fcimb.2014.00001)
Supplement: Supplementary file 4 [file DataSheet4.DOCX]

Supplementary Table 4. Genes showing ≥5-fold increase in expression (*P*< 0.05) during *in trans exsA* expression when compared to *in trans exsD* expression (see Material and Methods).

| **Locus Tag** | **Gene** | **Putative Product** | **COG** | **Fold Change** | ***P*-value** |
| --- | --- | --- | --- | --- | --- |
| *vp0088* | - | hypothetical protein | COG0106E | 7.3 | 0.00 |
| *vp0089* | - | hypothetical protein | - | 5.3 | 0.00 |
| *vp0254* | - | hypothetical protein | - | 26.7 | 0.00 |
| *vp0375* | - | lipoprotein | - | 9.4 | 0.00 |
| *vp0451* | - | hypothetical protein | - | 5.1 | 0.01 |
| *vp0509* | xerD | site-specific tyrosine recombinase XerD | COG4974L | 8.8 | 0.00 |
| *vp0579* | - | copper homeostasis protein | COG3142P | 12.0 | 0.00 |
| *vp0623* | - | D-amino acid dehydrogenase small subunit | COG0665E | 5.3 | 0.00 |
| *vp0636* | - | outer membrane protein A | COG3637M | 20.8 | 0.00 |
| *vp0801* | - | hypothetical protein | - | 10.7 | 0.01 |
| *vp0946* | - | cysteine synthase/cystathionine beta-synthase family protein | COG0031E | 9.1 | 0.00 |
| *vp1259* | - | ATP-dependent helicase | COG1643L | 5.8 | 0.00 |
| *vp1386* | - | hypothetical protein | - | 6.7 | 0.00 |
| *vp1391* | - | transcriptional regulator | COG3604KT | 11.0 | 0.00 |
| *vp1392* | - | ClpA/B-type protease | COG0542O | 21.9 | 0.00 |
| *vp1393* | - | BfdA protein | COG3157S | 5.9 | 0.00 |
| *vp1400* | - | hypothetical protein | COG0515RTKL | 20.9 | 0.00 |
| *vp1401* | - | hypothetical protein | COG3515S | 24.6 | 0.00 |
| *vp1402* | - | hypothetical protein | COG3516S | 23.9 | 0.00 |
| *vp1403* | - | hypothetical protein | COG3517S | 22.0 | 0.00 |
| *vp1404* | - | hypothetical protein | COG3518S | 15.0 | 0.00 |
| *vp1405* | - | hypothetical protein | COG3519S | 19.7 | 0.00 |
| *vp1406* | - | hypothetical protein | COG3520S | 5.2 | 0.00 |
| *vp1407* | - | transcriptional regulator | COG1522K | 7.1 | 0.00 |
| *vp1408* | - | IcmF-like protein | COG3523S | 11.8 | 0.00 |
| *vp1409* | - | hypothetical protein | COG3515S | 15.5 | 0.00 |
| *vp1410* | - | hypothetical protein | - | 15.0 | 0.00 |
| *vp1411* | - | hypothetical protein | COG3456T | 13.8 | 0.00 |
| *vp1412* | - | hypothetical protein | COG3521S | 15.7 | 0.00 |
| *vp1413* | - | hypothetical protein | COG3522S | 15.2 | 0.00 |
| *vp1414* | - | hypothetical protein | COG3455S | 7.4 | 0.00 |
| *vp1480* | - | riboflavin synthase subunit alpha | COG0307H | 7.2 | 0.00 |
| *vp1656* | YopD homolog | hydrophobic translocator | - | 1127.8 | 0.00 |
| *vp1657* | YopB homolog | hydrophobic translocator | COG5613S | 719.7 | 0.00 |
| *vp1658* | LcrH homolog | class II translocator chaperone | COG5010U | 696.0 | 0.00 |
| *vp1659* | LcrV homolog | hydrophilic translocator, injectisome tip | - | 770.6 | 0.00 |
| *vp1660* | LcrG homolog | LcrV chaperone, negative regulator of effector secretion | - | 1949.8 | 0.00 |
| *vp1661* | LcrR homolog | regulator, low calcium response protein | - | 1522.3 | 0.00 |
| *vp1662* | YscV homolog | inner membrane export apparatus | COG4789U | 928.1 | 0.00 |
| *vp1663* | YscY homolog | putative YscX chaperone | COG4783R | 6477.2 | 0.00 |
| *vp1664* | YscX homolog | unknown | - | 20533.9 | 0.00 |
| *vp1665* | SycN homolog | YopN/SycN/YscB/TyeA complex | - | 1921.0 | 0.00 |
| *vp1666* | TyeA homolog | YopN/SycN/YscB/TyeA complex | - | 4415.3 | 0.00 |
| *vp1667* | YopN homolog | YopN/SycN/YscB/TyeA complex | - | 1477.3 | 0.00 |
| *vp1668* | YscN homolog | ATPase | COG1157NU | 658.1 | 0.00 |
| *vp1669* | YscO homolog | unknown | - | 2957.6 | 0.00 |
| *vp1670* | YscP homolog | ruler - needle length control, substrate specificity switch | - | 298.1 | 0.00 |
| *vp1671* | YscQ homolog | cytoplasmic ring - sorting platform for T3S cargo proteins | COG1886NU | 2768.2 | 0.00 |
| *vp1672* | YscR homolog | inner membrane export apparatus | COG4790U | 517.3 | 0.00 |
| *vp1673* | YscS homolog | inner membrane export apparatus | COG4794U | 469.0 | 0.00 |
| *vp1674* | YscT homolog | inner membrane export apparatus | COG4791U | 85.6 | 0.00 |
| *vp1675* | YscU homolog | inner membrane export apparatus | COG4792U | 38.4 | 0.00 |
| *vp1678* | - | putative dienelactone hydrolase and related enzymes | COG1073R | 9.5 | 0.00 |
| *vp1679* | - | hypothetical protein | COG0716C | 190.6 | 0.00 |
| *vp1680* | VopQ/VepA | autophagy effector protein | - | 5381.2 | 0.00 |
| *vp1682* | VopQ chaperone/VecA | VopQ chaperone | - | 10052.6 | 0.00 |
| *vp1683* | VopR | unknown – putative effector protein | - | 1281.5 | 0.00 |
| *vp1684* | - | CesT family chaperone | - | 126.8 | 0.00 |
| *vp1685* | - | hypothetical protein | - | 397.7 | 0.00 |
| *vp1686* | VopS | Rho GTPase inhibition effector protein, actin rearrangement | COG3177S | 3778.8 | 0.00 |
| *vp1687* | VopS chaperone | putative VopS chaperone | - | 8005.8 | 0.00 |
| *vp1688* | YscL homolog | interactor of ATPase/C ring | COG1317NU | 464.3 | 0.00 |
| *vp1689* | YscK homolog | interactor of ATPase/C ring | - | 406.2 | 0.00 |
| *vp1690* | YscJ homolog | membrane and supramembrane (MS) ring | COG4669U | 649.3 | 0.00 |
| *vp1691* | YscI homolog | inner rod protein | - | 1277.3 | 0.00 |
| *vp1692* | YscH homolog | encodes YopR - unknown function | - | 859.6 | 0.00 |
| *vp1693* | YscG homolog | class III chaperone | - | 835.0 | 0.00 |
| *vp1694* | YscF homolog | needle protein | - | 721.9 | 0.00 |
| *vp1695* | YscD homolog | membrane and supramembrane (MS) ring | - | 880.1 | 0.00 |
| *vp1696* | YscC homolog | outer membrane secretin ring | COG1450NU | 969.2 | 0.00 |
| *vp1697* | YscB homolog | YopN/SycN/YscB/TyeA complex | - | 2674.4 | 0.00 |
| *vp1699* | ExsA | positive regulator of T3SS1 activity | COG2207K | 9331.4 | 0.00 |
| *vp1700* | YscW homolog | pilotin lipoprotein | - | 107.2 | 0.00 |
| *vp1701* | ExsC | putative ExsD inhibitor | - | 338.5 | 0.00 |
| *vp1702* | ExsE | putative ExsC inhibitor | - | 274.7 | 0.00 |
| *vp1787* | - | transposase | COG2801L | 6.0 | 0.00 |
| *vp1788* | - | transposase | COG2963L | 6.6 | 0.00 |
| *vp1789* | - | hypothetical protein | COG1278K | 6.8 | 0.00 |
| *vp1790* | - | hypothetical protein | COG3791S | 6.7 | 0.00 |
| *vp1831* | - | hypothetical protein | COG1694R | 5.2 | 0.00 |
| *vp1913* | - | hypothetical protein | - | 7.4 | 0.00 |
| *vp1980* | - | hypothetical protein | COG1032C | 9.9 | 0.00 |
| *vp2044* | - | hypothetical protein | - | 8.6 | 0.00 |
| *vp2082* | - | acetate kinase | COG0282C | 6.9 | 0.00 |
| *vp2083* | - | phosphate acetyltransferase | COG0280C | 5.3 | 0.00 |
| *vp2100* | - | excinuclease ABC subunit B | COG0556L | 11.4 | 0.00 |
| *vp2128* | - | nucleoid-associated protein NdpA | COG3081R | 5.5 | 0.00 |
| *vp2377* | - | hypothetical protein | COG1275P | 13.5 | 0.00 |
| *vp2385* | - | glycerol uptake facilitator protein GlpF | COG0580G | 9.1 | 0.00 |
| *vp2389* | - | membrane transport protein | COG0598P | 8.9 | 0.00 |
| *vp2415* | - | hypothetical protein | - | 44.1 | 0.00 |
| *vp2416* | - | hypothetical protein | - | 63.5 | 0.02 |
| *vp2417* | - | hypothetical protein | COG4961U | 5.5 | 0.00 |
| *vp2418* | - | hypothetical protein | - | 26.9 | 0.00 |
| *vp2419* | - | pilus assembly transmembrane protein | COG4964U | 8.0 | 0.00 |
| *vp2420* | - | pilus assembly protein | COG3745U | 7.9 | 0.00 |
| *vp2421* | - | hypothetical protein | COG0606O | 9.3 | 0.00 |
| *vp2422* | - | hypothetical protein | COG4960OU | 24.2 | 0.00 |
| *vp2423* | - | fimbrial protein | COG3847U | 45.1 | 0.00 |
| *vp2594* | - | hypothetical protein | COG2968S | 7.6 | 0.00 |
| *vp2596* | - | LysE/YggA family protein | COG1279R | 15.2 | 0.00 |
| *vp2597* | - | DNA-binding protein | COG2606S | 12.0 | 0.00 |
| *vp2743* | - | DamX-like protein | COG3266S | 5.4 | 0.00 |
| *vp2744* | aroB | 3-dehydroquinate synthase | COG0337E | 5.5 | 0.00 |
| *vp2758* | - | acetylglutamate kinase | COG0548E | 6.1 | 0.00 |
| *vp2849* | - | hypothetical protein | COG1285S | 6.1 | 0.00 |
| *vpa0006* | - | GTP cyclohydrolase II | COG0807H | 5.3 | 0.00 |
| *vpa0039* | - | hypothetical protein | - | 5.3 | 0.00 |
| *vpa0159* | - | hypothetical protein | COG5266P | 5.2 | 0.00 |
| *vpa0168* | emrD | multidrug resistance protein D | COG2814G | 5.6 | 0.00 |
| *vpa0194* | - | hypothetical protein | - | 16.9 | 0.00 |
| *vpa0221* | - | carbonic anhydrase | COG3338P | 5.9 | 0.00 |
| *vpa0252* | - | hypothetical protein | - | 20.2 | 0.00 |
| *vpa0289* | - | hypothetical protein | COG1280E | 15.6 | 0.02 |
| *vpa0303* | - | regulatory protein | COG1349KG | 8.8 | 0.00 |
| *vpa0304* | - | hypothetical protein | COG3324R | 28.4 | 0.00 |
| *vpa0312* | - | hypothetical protein | COG3134S | 49.9 | 0.00 |
| *vpa0319* | - | hypothetical protein | - | 13.9 | 0.00 |
| *vpa0320* | - | lipoprotein | COG3015MP | 14.9 | 0.00 |
| *vpa0405* | - | hypothetical protein | COG3608R | 5.1 | 0.00 |
| *vpa0450* | VPA0450 | inositol phosphatase effector protein | - | 2125.7 | 0.00 |
| *vpa0451* | VPA0450 chaperone | putative VPA0450 chaperone | - | 931.1 | 0.00 |
| *vpa0471* | - | multidrug efflux membrane fusion protein | COG0841V | 28.6 | 0.00 |
| *vpa0487* | - | hypothetical protein | - | 8.9 | 0.00 |
| *vpa0510* | - | hypothetical protein | COG4531P | 12.1 | 0.00 |
| *vpa0596* | - | methyl-accepting chemotaxis protein | COG0840NT | 41.4 | 0.00 |
| *vpa0597* | - | hypothetical protein | - | 42.8 | 0.00 |
| *vpa0599* | - | LysR family transcriptional regulator | COG0583K | 6.1 | 0.00 |
| *vpa0635* | - | oxidoreductase, oxygen dependent, FAD-dependent protein | COG0277C | 6.3 | 0.02 |
| *vpa0670* | - | hypothetical protein | - | 11.7 | 0.00 |
| *vpa0699* | - | hypothetical protein | COG4783R | 20.3 | 0.00 |
| *vpa0740* | - | transcriptional regulator | COG0583K | 7.5 | 0.00 |
| *vpa0747* | - | MSHA pilin protein MshA | COG2165NU | 8.3 | 0.00 |
| *vpa0788* | - | outer membrane phospholipase A | COG2829M | 10.0 | 0.00 |
| *vpa0882* | - | heme transport protein HutA | COG1629P | 7.6 | 0.00 |
| *vpa0924* | - | hypothetical protein | - | 6.3 | 0.00 |
| *vpa0984* | - | uroporphyrin-III C-methyltransferase | COG0007H | 7.0 | 0.00 |
| *vpa0991* | - | hypothetical protein | COG1470S | 5.4 | 0.00 |
| *vpa1021* | - | hypothetical protein | - | 14.4 | 0.00 |
| *vpa1140* | - | hypothetical protein | COG0384R | 7.2 | 0.00 |
| *vpa1239* | - | ABC transporter | COG0661R | 12.5 | 0.00 |
| *vpa1244* | - | hypothetical protein | - | 8.6 | 0.00 |
| *vpa1275* | - | short chain dehydrogenase/reductase family oxidoreductase | COG4221R | 10.5 | 0.00 |
| *vpa1278* | - | hypothetical protein | COG3918S | 5.8 | 0.00 |
| *vpa1297* | - | hypothetical protein | - | 49.5 | 0.04 |
| *vpa1343* | - | hypothetical protein | - | 5.8 | 0.00 |
| *vpa1356* | - | hypothetical protein | - | 8.7 | 0.00 |
| *vpa1424* | - | PTS system fructose-specific transporter subunit IIABC | COG1299G | 82.4 | 0.03 |
| *vpa1500* | - | hypothetical protein | - | 10.2 | 0.00 |
| *vpa1549* | - | hypothetical protein | - | 8.6 | 0.00 |
| *vpa1558* | - | hypothetical protein | - | 8.6 | 0.00 |
| *vpa1559* | - | hypothetical protein | - | 8.9 | 0.00 |
