# Supplementary material for: Transcriptome analysis of Vibrio parahaemolyticus in type III secretion system 1 inducing conditions
Source: Front Cell Infect Microbiol. 2014 Jan 20;4:1. doi: 10.3389/fcimb.2014.00001 (PMC3895804; doi:10.3389/fcimb.2014.00001)
Supplement: Supplementary file 5 [file DataSheet5.DOCX]

Supplementary Table 5. Genes showing ≥150-fold change (*P*< 0.05) in expression at 2 hr post-infection of HeLa cells compared to 0 hr (pre-infection).

| **Locus Tag** | **Putative Product** | **COG** | **2 Hr Fold Change** |
| --- | --- | --- | --- |
| *vpa0633* | hypothetical protein | - | 714.5 |
| *vpa1658* | hypothetical protein | COG0439I | 606.3 |
| *vpa0632* | hypothetical protein | - | 503.6 |
| *vpa0422* | hemin ABC transporter permease | COG0609P | 474.2 |
| *vpa1660* | transport protein | COG2814G | 428.8 |
| *vpa0882* | heme transport protein HutA | COG1629P | 412.6 |
| *vpa0421* | hemin importer ATP-binding subunit | COG4559P | 365.7 |
| *vpa1656* | ferric vibrioferrin receptor | COG4772P | 312.4 |
| *vpa1661* | AcsD | COG4264Q | 280.2 |
| *vpa1659* | hypothetical protein | COG4264Q | 251.4 |
| *vpa0423* | hemin ABC transporter periplasmic hemin-binding protein HutB | COG4558P | 250.4 |
| *vpa0979* | ferric aerobactin receptor | COG1629P | 213.9 |
| *vpa1657* | ferric siderophore receptor-like protein | COG4774P | 194.2 |
| *vpa1653* | ferrichrome ABC transporter permease | COG0609P | 191.0 |
| *vp1974* | 5-methyltetrahydropteroyltriglutamate--homocysteine S-methyltransferase | COG0620E | 189.3 |
| *vpa0424* | TonB system transport protein ExbD1 | COG0848U | 186.3 |
| *vpa1466* | TonB system receptor | COG4771P | 181.3 |
| *vpa0425* | ExbB-like protein | COG0811U | 178.8 |
| *vpa0427* | coproporphyrinogen III oxidase | COG0635H | 166.9 |
| *vpa1655* | iron-dicitrate transporter substrate-binding subunit | COG4594P | 152.3 |
| *vp1164* | hypothetical protein | - | 151.0 |
