# Supplementary material for: Transcriptome analysis of Vibrio parahaemolyticus in type III secretion system 1 inducing conditions
Source: Front Cell Infect Microbiol. 2014 Jan 20;4:1. doi: 10.3389/fcimb.2014.00001 (PMC3895804; doi:10.3389/fcimb.2014.00001)
Supplement: Supplementary file 6 [file DataSheet6.DOCX]

Supplementary Table 6. Genes showing ≥150-fold change (*P*< 0.05) in expression at 3, 4, 6, or 8 hr post-infection of HeLa cells compared to 0 hr (pre-infection). NS: Not Significant (*P*> 0.05)

| **Feature ID** | **Gene** | **Product** | **COG** | **3 Hr Fold**  **Change** | **4 Hr Fold**  **Change** | **6 Hr Fold**  **Change** | **8 Hr Fold**  **Change** |
| --- | --- | --- | --- | --- | --- | --- | --- |
| *vpa1059* | - | hypothetical protein | - | 9059.8 | 6369.5 | 8514.2 | 6983.9 |
| *vpa1058* | - | nitrate transport protein | COG0715P | 6682.8 | 5548.9 | 7408.1 | 6137.8 |
| *vpa1057* | - | nitrate transporter | COG0600P | 1057.6 | 989.8 | 1082.6 | NS |
| *vpa1065* | - | nitrate reductase, large subunit protein | COG0243C | 918.4 | 1131.2 | 1321.8 | 1189.0 |
| *vp1621* | - | amino acid ABC transporter permease | COG4597E | 875.2 | 900.8 | NS | 478.0 |
| *vp1620* | - | amino acid ABC transporter substrate-binding protein | COG0834ET | 589.4 | 794.0 | 370.4 | 365.7 |
| *vpa0633* | - | hypothetical protein | - | 505.5 | NS | NS | 51.2 |
| *vpa1064* | - | siroheme synthase | COG0007H | 474.8 | 599.5 | 534.6 | 497.0 |
| *vpa1056* | - | nitrate transporter ATPase component NasD | COG1116P | 447.0 | NS | 419.6 | NS |
| *vp1622* | - | amino acid ABC transporter permease | COG0765E | 333.2 | 280.9 | 149.0 | 150.8 |
| *vpa0422* | - | hemin ABC transporter permease | COG0609P | 276.6 | 258.8 | 156.7 | 46.9 |
| *vpa0007* | - | hypothetical protein | - | NS | NS | 252.6 | 99.8 |
| *vpa0632* | - | hypothetical protein | - | 250.2 | 120.6 | 53.9 | 75.0 |
| *vp2070* | - | hypothetical protein | - | 62.9 | 70.2 | 87.1 | 246.1 |
| *vp1779* | - | glutamine amidotransferase | COG2071R | 241.6 | 111.2 | NS | 38.1 |
| *vpa1658* | - | hypothetical protein | COG0439I | 228.0 | 122.4 | 50.0 | 18.7 |
| *vp1663* | YscY homolog | putative YscX chaperone | COG4783R | 201.8 | 336.2 | 371.2 | 428.1 |
| *vpa0421* | hmuV | hemin importer ATP-binding subunit | COG4559P | 198.9 | 188.7 | 86.2 | 23.4 |
| *vpa0423* | - | hemin ABC transporter periplasmic hemin-binding protein HutB | COG4558P | 189.8 | 162.0 | 96.1 | 35.9 |
| *vp1673* | YscS homolog | inner Membrane export apparatus | COG4794U | 174.3 | 243.1 | 237.6 | 305.0 |
| *vpa1055* | - | nitrite reductase | COG1251C | 159.7 | 128.8 | 118.7 | NS |
| *vpa1656* | - | ferric vibrioferrin receptor | COG4772P | 159.1 | 98.3 | 53.4 | 25.2 |
| *vpa0424* | - | TonB system transport protein ExbD1 | COG0848U | 155.1 | 149.9 | 96.7 | 33.0 |
| *vp1674* | YscT homolog | inner membrane export apparatus | COG4791U | 151.3 | 217.6 | 172.7 | 210.0 |
| *vp1657* | YopB homolog | hydrophobic translocator | COG5613S | 146.8 | 204.4 | 193.5 | 452.9 |
| *vp1671* | YscQ homolog | cytoplasmic ring - sorting platform for T3S cargo proteins | COG1886NU | 143.4 | 200.4 | 220.7 | 290.5 |
| *vp1672* | YscR homolog | inner membrane export apparatus | COG4790U | 134.1 | 184.0 | 169.9 | 226.0 |
| *vpa1060* | - | two-component response regulatory protein | COG3707T | 122.1 | 103.7 | 151.5 | 178.4 |
| *vp1656* | YopD homolog | hydrophobic translocator | - | 116.0 | NS | 153.5 | 322.7 |
| *vp1658* | LcrH homolog | class II translocator chaperone | COG5010U | 115.4 | 149.0 | 165.9 | 170.4 |
| *vp1695* | YscD homolog | membrane and supramembrane (MS) ring | - | 109.8 | 155.3 | 176.4 | 213.0 |
| *vp1662* | YscV homolog | inner membrane export apparatus | COG4789U | 107.1 | 157.1 | 157.4 | 180.3 |
| *vp1670* | YscP homolog | ruler - needle length control, substrate specificity switch | - | 104.1 | 162.8 | 195.4 | 235.1 |
| *vpa0450* | VPA0450 | inositol phosphatase effector protein | - | 91.6 | 155.6 | 176.3 | 170.9 |
| *vp1696* | YscC homolog | outer membrane secretin ring | COG1450NU | 79.7 | 144.1 | 142.5 | 165.8 |
| *vp1664* | YscX homolog | unknown | - | 79.0 | 140.8 | 191.7 | 198.3 |
| *vp1697* | YscB homolog | YopN/SycN/YscB/TyeA complex | - | 71.9 | 131.7 | 148.0 | 157.8 |
