# Supplementary material for: Transcriptome analysis of Vibrio parahaemolyticus in type III secretion system 1 inducing conditions
Source: Front Cell Infect Microbiol. 2014 Jan 20;4:1. doi: 10.3389/fcimb.2014.00001 (PMC3895804; doi:10.3389/fcimb.2014.00001)
Supplement: Supplementary file 7 [file DataSheet7.DOCX]

Supplementary Table 7. (A) Genes showing upregulation over the course of HeLa cell infection compared to 0 hr (pre-infection), defined as increasing fold change from 3 hr – 6 hr post-infection, ≥10-fold increase between 2 hr and 8 hr post-infection and 8 hr fold change >0 (*P*< 0.05). (B) Genes showing downregulation over the course of HeLa cell infection compared to 0 hr (pre-infection), defined as decreasing fold change from 3 hr – 6 hr post-infection and ≥10-fold decrease between 2 hr and 8 hr post-infection (*P*< 0.05). (C) Genes generally upregulated but relatively static over the course of HeLa cell infection compared to 0 hr (pre-infection), defined as ≥5-fold change at 2 hr post-infection ± 3-fold change at 3 hr – 8 hr compared to the previous time point (*P*< 0.05).

(A)

| **Locus Tag** | **Gene** | **Putative Product** | **COG** | **2 Hr Fold**  **Change** | **3 Hr Fold**  **Change** | **4 Hr Fold**  **Change** | **6 Hr Fold**  **Change** | **8 Hr Fold**  **Change** |
| --- | --- | --- | --- | --- | --- | --- | --- | --- |
| *vp0018* | - | 16 kDa heat shock protein A | COG0071O | 1.7 | 2.5 | 4.6 | 15.3 | 12.6 |
| *vp0481* | gltD | glutamate synthase subunit beta | COG0493ER | 7.6 | 14.3 | 18.0 | 20.7 | 32.4 |
| *vp0483* | gltD | glutamate synthase subunit beta | COG0493ER | 44.4 | 48.8 | 69.4 | 88.9 | 130.2 |
| *vp0985* | - | SpoVR family protein | COG2719S | 3.1 | 9.8 | 12.9 | 18.0 | 32.1 |
| *vp0986* | - | hypothetical protein | COG2718S | 2.1 | 11.3 | 15.3 | 25.5 | 40.7 |
| *vp0987* | - | hypothetical protein | - | 2.4 | 9.4 | 12.7 | 25.1 | 29.1 |
| *vp1012* | - | cold shock-like protein CspD | COG1278K | 7.3 | 21.6 | 31.9 | 42.5 | 70.0 |
| *vp1205* | - | hypothetical protein | - | 1.9 | 3.9 | 5.2 | 9.3 | 13.9 |
| *vp1379* | - | homoserine/homoserine lactone efflux protein | COG1280E | 3.8 | 12.8 | 15.1 | 19.1 | 22.1 |
| *vp1605* | - | hypothetical protein | - | 3.9 | 4.0 | 7.8 | 19.2 | 39.2 |
| *vp1658* | LcrH homolog | class II translocator chaperone | COG5010U | 70.5 | 115.4 | 149.0 | 165.9 | 170.4 |
| *vp1659* | LcrV homolog | hydrophilic translocator, Injectisome Tip | - | 20.4 | 51.4 | 96.0 | 111.7 | 109.3 |
| *vp1660* | LcrG homolog | LcrV chaperone, negative regulator of effector secretion | - | 19.7 | 50.4 | 81.3 | 104.6 | 104.3 |
| *vp1661* | LcrR homolog | regulator, low calcium response protein | - | 58.6 | 87.3 | 125.5 | 134.1 | 142.6 |
| *vp1662* | YscV homolog | inner membrane export apparatus | COG4789U | 63.0 | 107.1 | 157.1 | 157.4 | 180.3 |
| *vp1663* | YscY homolog | putative YscX chaperone | COG4783R | 104.0 | 201.8 | 336.2 | 371.2 | 428.1 |
| *vp1664* | YscX homolog | unknown | - | 31.8 | 79.0 | 140.8 | 191.7 | 198.3 |
| *vp1665* | SycN homolog | YopN/SycN/YscB/TyeA complex | - | 15.0 | 36.9 | 63.0 | 94.9 | 113.0 |
| *vp1666* | TyeA homolog | YopN/SycN/YscB/TyeA complex | - | 12.3 | 35.0 | 69.4 | 96.0 | 97.0 |
| *vp1667* | YopN homolog | YopN/SycN/YscB/TyeA complex | - | 9.0 | 24.3 | 46.0 | 60.7 | 66.2 |
| *vp1668* | YscN homolog | ATPase | COG1157NU | 13.4 | 29.1 | 53.7 | 65.3 | 89.0 |
| *vp1669* | YscO homolog | unknown | - | 15.1 | 46.6 | 72.2 | 100.5 | 111.7 |
| *vp1670* | YscP homolog | ruler - needle length control, substrate specificity switch | - | 49.0 | 104.1 | 162.8 | 195.4 | 235.1 |
| *vp1671* | YscQ homolog | cytoplasmic ring - sorting platform for T3S cargo proteins | COG1886NU | 93.1 | 143.4 | 200.4 | 220.7 | 290.5 |
| *vp1682* | VopQ chaperone/VecA | VopQ chaperone | - | 6.8 | 26.6 | 43.7 | 64.5 | 70.3 |
| *vp1683* | VopR | unknown - putative effector protein | - | 8.8 | 15.9 | 24.4 | 32.7 | 30.4 |
| *vp1684* | CesT family chaperone | unknown | - | 5.9 | 12.1 | 18.4 | 26.6 | 27.5 |
| *vp1685* | Hypothetical protein | unknown | - | 8.9 | 22.9 | 40.2 | 56.1 | 66.6 |
| *vp1686* | VopS | Rho GTPase inhibition effector protein, actin rearrangement | COG3177S | 9.5 | 29.8 | 57.0 | 80.0 | 67.9 |
| *vp1687* | VopS chaperone | putative VopS chaperone | - | 10.2 | 28.9 | 60.8 | 85.0 | 81.0 |
| *vp1692* | YscH homolog | encodes YopR - unknown function | - | 65.8 | 65.8 | 78.4 | 82.2 | 87.0 |
| *vp1695* | YscD homolog | membrane and supramembrane (MS) ring | - | 89.0 | 109.8 | 155.3 | 176.4 | 213.0 |
| *vp1697* | YscB homolog | YopN/SycN/YscB/TyeA complex | - | 34.7 | 71.9 | 131.7 | 148.0 | 157.8 |
| *vp1698* | ExsD | negative regulator of T3SS1 activity | - | 21.1 | 47.3 | 76.0 | 79.4 | 99.0 |
| *vp1699* | ExsA | positive regulator of T3SS1 activity | COG2207K | 13.2 | 15.2 | 18.2 | 19.6 | 24.5 |
| *vp1700* | YscW homolog | pilotin lipoprotein | - | 5.0 | 12.1 | 15.0 | 19.3 | 18.3 |
| *vp1702* | ExsE | putative ExsC inhibitor | - | 20.0 | 37.5 | 50.8 | 54.1 | 105.7 |
| *vp2070* | - | hypothetical protein | - | 8.6 | 62.9 | 70.2 | 87.1 | 246.1 |
| *vp2371* | - | N-acetylglutamate synthase | COG0548E | 5.6 | 8.2 | 8.7 | 16.5 | 16.4 |
| *vp2553* | - | RNA polymerase sigma factor RpoS | COG0568K | -1.6 | 2.1 | 3.1 | 5.2 | 10.5 |
| *vp2756* | - | bifunctional argininosuccinate lyase/N-acetylglutamate synthase | COG0165E | 7.5 | 13.1 | 14.4 | 23.4 | 24.6 |
| *vp2757* | - | argininosuccinate synthase | COG0137E | 12.4 | 29.2 | 33.1 | 52.5 | 65.4 |
| *vp2758* | - | acetylglutamate kinase | COG0548E | 9.5 | 21.3 | 22.6 | 43.7 | 45.7 |
| *vp2910* | - | hypothetical protein | COG4974L | 2.0 | 4.8 | 6.7 | 11.6 | 15.1 |
| *vpa0035* | - | sodium/glutamate symporter | COG0786E | -7.0 | 1.3 | 1.7 | 3.0 | 3.5 |
| *vpa0450* | VPA0450 | inositol phosphatase effector protein | - | 29.4 | 91.6 | 155.6 | 176.3 | 170.9 |
| *vpa0451* | VPA0450 chaperone | putative VPA0450 chaperone | - | 11.9 | 43.4 | 71.0 | 129.5 | 141.7 |
| *vpa0639* | artM | arginine transporter permease subunit ArtM | COG4160E | 40.5 | 90.1 | 113.9 | 124.2 | 57.8 |
| *vpa0669* | - | hypothetical protein | - | 2.0 | 4.1 | 6.2 | 10.1 | 12.4 |
| *vpa0680* | - | arylsulfatase | COG3119P | 6.9 | 18.4 | 25.9 | 40.5 | 59.9 |
| *vpa0694* | - | hypothetical protein | COG3111S | 2.1 | 3.5 | 5.6 | 14.7 | 14.2 |
| *vpa1050* | - | hypothetical protein | - | 6.0 | 15.6 | 20.4 | 36.7 | 50.7 |
| *vpa1186* | - | outer membrane protein OmpA | COG2885M | 4.5 | 14.3 | 18.4 | 19.6 | 38.2 |
| *vpa1287* | - | transporter | COG1230P | 2.2 | 5.9 | 7.3 | 12.2 | 18.2 |
| *vpa1387* | - | hypothetical protein | - | 2.8 | 7.5 | 11.9 | 15.3 | 18.8 |
| *vpa1388* | - | hypothetical protein | - | 6.2 | 12.7 | 18.1 | 21.3 | 28.1 |
| *vpa1391* | - | hypothetical protein | COG1476K | 2.4 | 5.3 | 6.8 | 10.3 | 17.5 |
| *vpa1394* | - | transposition protein | COG1474LO | 4.4 | 10.3 | 13.2 | 17.1 | 28.2 |
| *vpa1395* | - | transposase | - | 5.7 | 18.1 | 25.9 | 37.5 | 63.1 |
| *vpa1396* | - | hypothetical protein | - | 2.3 | 6.9 | 11.4 | 15.9 | 33.5 |
| *vpa1398* | - | hypothetical protein | - | 4.0 | 7.4 | 10.9 | 16.6 | 26.3 |
| *vpa1446* | - | LuxR family transcriptional regulator | COG2197TK | 5.8 | 12.0 | 14.5 | 15.0 | 18.2 |

(B)

| **Locus Tag** | **Gene** | **Putative Product** | **COG** | **2 Hr Fold**  **Change** | **3 Hr Fold**  **Change** | **4 Hr Fold**  **Change** | **6 Hr Fold**  **Change** | **8 Hr Fold**  **Change** |
| --- | --- | --- | --- | --- | --- | --- | --- | --- |
| *vp0004* | rnpA | ribonuclease P | COG0594J | 14.1 | 7.1 | 5.8 | 3.5 | 3.3 |
| *vp0053* | - | hypothetical protein | - | -8.5 | -41.2 | -44.5 | -65.1 | -43.2 |
| *vp0055* | - | RNA polymerase ECF-type sigma factor | COG1595K | -1.4 | -4.7 | -6.6 | -11.5 | -12.1 |
| *vp0244* | glpX | fructose 1,6-bisphosphatase II | COG1494G | -1.7 | -11.9 | -13.3 | -15.7 | -13.9 |
| *vp0406* | - | hypothetical protein | COG1610S | 15.7 | 8.1 | 5.6 | 3.3 | 2.6 |
| *vp0407* | rpsU | 30S ribosomal protein S21 | COG0828J | 13.7 | 5.1 | 3.8 | 2.7 | 1.8 |
| *vp0712* | - | hypothetical protein | - | -27.0 | -53.3 | -66.2 | -79.0 | -81.8 |
| *vp1008* | - | outer membrane porin protein | COG3203M | 71.1 | 40.0 | 20.4 | 13.7 | 13.5 |
| *vp1317* | - | glutaredoxin | COG0695O | -4.0 | -4.0 | -5.3 | -21.1 | -18.4 |
| *vp1917* | - | hypothetical protein | COG3490S | 68.3 | 32.3 | 26.7 | 20.5 | 15.7 |
| *vp1918* | - | hypothetical protein | COG3489R | 52.9 | 29.6 | 23.0 | 15.7 | 14.5 |
| *vp1919* | - | hypothetical protein | COG3488C | 84.3 | 53.9 | 40.5 | 28.7 | 24.7 |
| *vp1920* | - | iron-regulated protein A | COG3487P | 79.6 | 58.4 | 40.6 | 20.9 | 17.9 |
| *vp1991* | - | 5-methyltetrahydropteroyltriglutamate--homocysteine S-methyltransferase | COG0620E | 113.4 | 45.6 | 20.6 | 14.9 | 25.8 |
| *vp1992* | - | hypothetical protein | - | 116.7 | 74.5 | 34.1 | 23.2 | 41.8 |
| *vp2161* | - | hypothetical protein | COG3016S | 48.8 | 25.0 | 19.7 | 8.6 | 4.2 |
| *vp2167* | - | hypothetical protein | COG1359S | -1.3 | -5.7 | -18.0 | -20.2 | -19.8 |
| *vp2329* | - | efflux pump component MtrF | COG2978H | -6.7 | -9.2 | -12.6 | -28.9 | -22.4 |
| *vp2362* | - | outer membrane protein OmpK | COG3248M | -5.1 | -36.6 | -58.3 | -74.9 | -93.4 |
| *vp2437* | - | NupC family protein | COG1972F | -1.4 | -5.4 | -8.2 | -11.1 | -12.6 |
| *vp2491* | - | iron(III) ABC transporter periplasmic iron-compound-binding protein | COG1840P | 30.8 | 18.2 | 17.1 | 14.6 | 11.5 |
| *vp2861* | - | rRNA methylase | COG0219J | 14.2 | 9.6 | 6.9 | 4.1 | 2.9 |
| *vp2873* | fumC | fumarate hydratase | COG0114C | 13.4 | 11.3 | 9.3 | 5.4 | 2.4 |
| *vp2930* | tuf | elongation factor Tu | COG0050J | -2.2 | -7.2 | -10.1 | -12.0 | -14.7 |
| *vpa0005* | - | hypothetical protein | - | -5.9 | -6.2 | -10.8 | -17.4 | -23.2 |
| *vpa0149* | - | two-component system sensor kinase | COG0642T | 13.7 | 4.8 | 4.1 | 2.4 | 1.9 |
| *vpa0153* | - | TonB system transport protein ExbB2 | COG0811U | 12.5 | 8.7 | 6.4 | 4.3 | 1.9 |
| *vpa0155* | - | TonB2 protein | COG0810M | 12.7 | 10.0 | 8.1 | 5.1 | 2.0 |
| *vpa0156* | - | hypothetical protein | COG2956G | 19.9 | 14.0 | 12.3 | 6.7 | 3.7 |
| *vpa0226* | - | lecithin-dependent hemolysin (LDH) | COG3240IR | 16.8 | 10.3 | 7.2 | 6.0 | 5.0 |
| *vpa0421* | hmuV | hemin importer ATP-binding subunit | COG4559P | 365.7 | 198.9 | 188.7 | 86.2 | 23.4 |
| *vpa0422* | - | hemin ABC transporter permease | COG0609P | 474.1 | 276.6 | 258.8 | 156.7 | 46.9 |
| *vpa0423* | - | hemin ABC transporter periplasmic hemin-binding protein HutB | COG4558P | 250.4 | 189.8 | 162.0 | 96.1 | 35.9 |
| *vpa0424* | - | TonB system transport protein ExbD1 | COG0848U | 186.3 | 155.1 | 149.9 | 96.7 | 33.0 |
| *vpa0429* | - | hypothetical protein | COG0748P | 31.2 | 18.8 | 12.4 | 7.5 | 3.1 |
| *vpa0430* | - | hypothetical protein | COG2096S | 16.7 | 8.1 | 6.1 | 4.3 | 3.4 |
| *vpa0627* | - | cytochrome o ubiquinol oxidase subunit II | COG1622C | 37.1 | 17.5 | 11.6 | 8.8 | 5.1 |
| *vpa0628* | - | cytochrome o ubiquinol oxidase subunit I | COG0843C | 36.5 | 16.5 | 10.8 | 8.0 | 5.1 |
| *vpa0629* | - | cytochrome o ubiquinol oxidase subunit III | COG1845C | 29.6 | 15.1 | 9.9 | 6.7 | 4.8 |
| *vpa0630* | - | hypothetical protein | COG3125C | 20.2 | 11.2 | 6.7 | 3.8 | 2.9 |
| *vpa0631* | - | protoheme IX farnesyltransferase | COG0109O | 40.0 | 17.7 | 12.0 | 8.1 | 6.7 |
| *vpa0632* | - | hypothetical protein | - | 503.6 | 250.2 | 120.6 | 53.9 | 75.0 |
| *vpa0658* | - | iron(III) ABC transporter permease | COG4606P | 123.7 | 95.8 | 88.6 | 48.1 | 25.7 |
| *vpa0659* | - | iron(III) ABC transporter permease | COG4605P | 84.7 | 57.4 | 49.9 | 24.7 | 12.2 |
| *vpa0660* | - | iron(III) ABC transporter ATP-binding protein | COG4604P | 34.5 | 19.2 | 18.4 | 9.9 | 5.3 |
| *vpa0802* | - | glycine cleavage system protein H | COG0509E | -3.8 | -5.1 | -9.0 | -18.1 | -21.2 |
| *vpa0882* | - | heme transport protein HutA | COG1629P | 412.6 | 121.6 | 70.9 | 21.5 | 5.1 |
| *vpa0979* | - | ferric aerobactin receptor | COG1629P | 213.8 | 85.7 | 62.6 | 27.0 | 7.3 |
| *vpa0980* | - | hypothetical protein | COG4114R | 66.9 | 58.1 | 55.8 | 23.2 | 15.4 |
| *vpa1401* | malE | maltose ABC transporter periplasmic protein | COG2182G | -22.3 | -46.1 | -83.4 | -98.6 | -58.5 |
| *vpa1435* | - | iron(III) compound receptor | COG4773P | 49.1 | 29.4 | 28.4 | 20.3 | 12.4 |
| *vpa1436* | - | iron(III) ABC transporter ATP-binding protein | COG1120PH | 88.3 | 55.1 | 41.4 | 26.8 | 18.0 |
| *vpa1437* | - | iron(III) ABC transporter periplasmic iron-compound-binding protein | COG0614P | 141.0 | 84.7 | 71.1 | 45.2 | 27.7 |
| *vpa1438* | - | iron-hydroxamate transporter permease subunit | COG0609P | 73.5 | 39.6 | 36.9 | 18.6 | 10.9 |
| *vpa1495* | - | ABC transporter ATP-binding protein | COG4172R | 124.9 | 77.2 | 47.7 | 16.2 | 3.3 |
| *vpa1609* | - | proton/glutamate symporter | COG1301C | -2.6 | -32.4 | -39.4 | -42.9 | -42.8 |
| *vpa1636* | - | hypothetical protein | - | -3.4 | -5.0 | -8.7 | -9.3 | -17.1 |
| *vpa1639* | - | secreted ribonuclease | COG2356L | -2.2 | -6.3 | -11.7 | -14.5 | -15.0 |
| *vpa1650* | - | insulinase family zinc protease | COG0612R | 26.0 | 19.6 | 14.0 | 10.0 | 6.7 |
| *vpa1653* | - | ferrichrome ABC transporter permease | COG0609P | 191.0 | 118.1 | 87.7 | 40.9 | 17.9 |
| *vpa1654* | - | ferrichrome ABC transporter permease | COG0609P | 94.4 | 61.9 | 46.5 | 28.1 | 8.3 |
| *vpa1655* | fecB | iron-dicitrate transporter substrate-binding subunit | COG4594P | 152.3 | 98.5 | 70.3 | 39.7 | 18.4 |
| *vpa1656* | - | ferric vibrioferrin receptor | COG4772P | 312.4 | 159.1 | 98.3 | 53.4 | 25.2 |
| *vpa1657* | - | ferric siderophore receptor-like protein | COG4774P | 194.2 | 115.2 | 87.9 | 55.8 | 31.1 |
| *vpa1658* | - | hypothetical protein | COG0439I | 606.3 | 228.0 | 122.4 | 50.0 | 18.7 |
| *vpa1659* | - | hypothetical protein | COG4264Q | 251.4 | 70.3 | 31.9 | 14.2 | 4.0 |
| *vpa1660* | - | transport protein | COG2814G | 428.8 | 108.8 | 53.2 | 21.1 | 7.4 |
| *vpa1673* | - | L-arabinose-binding periplasmic protein | COG1879G | -77.6 | -88.6 | -138.6 | -162.7 | -172.5 |
| *vpa1681* | - | organic hydroperoxide resistance protein | COG1764O | -3.4 | -10.1 | -10.9 | -12.2 | -19.6 |

(C)

| **Locus Tag** | **Gene** | **Putative Product** | **COG** | **2 Hr Fold**  **Change** | **3 Hr Fold**  **Change** | **4 Hr Fold**  **Change** | **6 Hr Fold**  **Change** | **8 Hr Fold**  **Change** |
| --- | --- | --- | --- | --- | --- | --- | --- | --- |
| *vp0049* | - | peptide ABC transporter permease | COG0601EP | 8.6 | 10.8 | 9.3 | 8.3 | 6.4 |
| *vp0217* | - | regulator | - | 5.2 | 2.8 | 2.3 | 1.9 | 1.9 |
| *vp0220* | - | OtnA protein | COG1596M | 5.5 | 4.2 | 3.2 | 2.7 | 3.3 |
| *vp0378* | - | hypothetical protein | - | 5.5 | 6.2 | 5.3 | 3.6 | 2.6 |
| *vp0379* | - | ABC transporter substrate binding protein | COG1464P | 7.9 | 7.5 | 7.1 | 5.7 | 7.3 |
| *vp0548* | - | ToxR-activated protein TagE | COG0739M | 6.7 | 3.9 | 3.8 | 3.2 | 3.1 |
| *vp0618* | - | hypothetical protein | COG1757C | 5.7 | 6.8 | 6.9 | 9.8 | 10.1 |
| *vp1190* | - | anaerobic nitric oxide reductase transcription regulator | COG3604KT | 6.4 | 5.0 | 5.4 | 4.6 | 4.2 |
| *vp1215* | - | ribosomal small subunit pseudouridine synthase A | COG1187J | 5.3 | 3.4 | 2.8 | 1.5 | 1.7 |
| *vp1225* | - | ABC transporter ATP-binding protein | COG1136V | 8.1 | 8.1 | 8.2 | 5.5 | 3.9 |
| *vp1385* | - | hypothetical protein | COG0739M | 9.2 | 9.3 | 11.3 | 8.4 | 6.5 |
| *vp1386* | - | hypothetical protein | - | 5.4 | 5.0 | 5.7 | 4.0 | 3.7 |
| *vp1421* | - | hypothetical protein | COG0861P | 5.5 | 8.3 | 5.7 | 3.7 | 4.7 |
| *vp1563* | - | hypothetical protein | COG1609K | 11.2 | 11.9 | 13.5 | 12.3 | 10.9 |
| *vp1655* | - | immunogenic protein | COG2358R | 12.0 | 14.4 | 13.3 | 14.3 | 13.1 |
| *vp1676* | - | putative LysR-family transcriptional regulator | COG0583K | 6.1 | 5.6 | 6.4 | 5.0 | 5.6 |
| *vp1717* | - | hypothetical protein | COG0842V | 5.7 | 6.4 | 6.0 | 6.8 | 7.2 |
| *vp1738* | - | hypothetical protein | - | 7.1 | 9.2 | 10.1 | 9.0 | 8.1 |
| *vp1815* | - | hypothetical protein | - | 8.4 | 8.8 | 6.0 | 4.0 | 3.5 |
| *vp1816* | - | Hit protein involved in cell-cycle regulation | COG0537FGR | 5.1 | 6.6 | 5.7 | 4.7 | 4.5 |
| *vp1817* | - | hypothetical protein | - | 7.4 | 8.9 | 6.9 | 5.4 | 4.3 |
| *vp1818* | - | hypothetical protein | - | 7.1 | 6.2 | 4.8 | 4.6 | 6.0 |
| *vp1826* | - | hypothetical protein | COG1502I | 6.2 | 6.1 | 4.6 | 3.0 | 2.4 |
| *vp1844* | - | hypothetical protein | COG3981R | 5.2 | 4.8 | 4.1 | 2.5 | 2.7 |
| *vp1850* | - | acetyltransferase | COG0456R | 5.9 | 3.8 | 2.4 | 2.7 | 2.4 |
| *vp1951* | - | pseudouridine synthase | COG1187J | 6.8 | 3.9 | 4.0 | 3.5 | 3.3 |
| *vp1994* | - | isochorismatase-like protein | COG1335Q | 13.1 | 11.1 | 11.2 | 9.5 | 10.8 |
| *vp1995* | - | ABC transporter ATP-binding protein | COG1136V | 13.2 | 12.3 | 11.8 | 10.0 | 10.5 |
| *vp1997* | - | hypothetical protein | COG0577V | 8.2 | 9.2 | 11.0 | 11.2 | 11.7 |
| *vp2312* | - | 1-deoxy-D-xylulose 5-phosphate reductoisomerase | COG0743I | 5.4 | 3.2 | 2.6 | 1.9 | 2.1 |
| *vp2313* | - | phosphatidate cytidylyltransferase | COG0575I | 6.1 | 4.7 | 4.6 | 4.0 | 4.9 |
| *vp2472* | - | multidrug resistance protein | COG0841V | 5.5 | 4.1 | 2.9 | 2.8 | 2.3 |
| *vp2473* | - | hypothetical protein | COG0845M | 5.7 | 4.6 | 3.8 | 2.6 | 2.8 |
| *vp2768* | - | bacterioferritin | COG2193P | 20.5 | 19.1 | 20.0 | 19.3 | 21.4 |
| *vpa0010* | - | hypothetical protein | - | 5.8 | 4.1 | 3.8 | 3.7 | 3.9 |
| *vpa0652* | - | hypothetical protein | COG2847S | 5.7 | 4.1 | 4.2 | 3.2 | 3.2 |
| *vpa0668* | - | hypothetical protein | COG2931Q | 5.5 | 3.3 | 2.8 | 2.3 | 2.2 |
| *vpa0957* | - | transporter binding protein | COG4533R | 6.4 | 8.2 | 9.7 | 9.4 | 11.3 |
| *vpa0958* | - | NADH oxidase | COG0607P | 13.9 | 13.6 | 10.7 | 9.9 | 12.0 |
| *vpa1289* | - | cold shock transcriptional regulator CspA | COG1278K | 12.8 | 9.8 | 9.0 | 7.3 | 6.8 |
| *vpa1354* | - | putative type III secretion system EscU protein | COG1377NU | 7.1 | 6.8 | 7.6 | 9.3 | 9.9 |
| *vpa1357* | VopV | F actin binding effector protein | - | 5.1 | 4.7 | 5.1 | 5.0 | 4.4 |
| *vpa1364* | - | hypothetical protein | - | 12.9 | 13.7 | 14.8 | 17.0 | 19.3 |
| *vpa1368* | - | hypothetical protein | - | 6.5 | 8.8 | 7.1 | 5.8 | 6.5 |
| *vpa1380* | - | OspB protein | - | 11.7 | 13.4 | 12.8 | 12.0 | 13.8 |
| *vpa1392* | - | hypothetical protein | - | 10.2 | 8.9 | 10.6 | 8.9 | 11.3 |
